# Supplementary material for: Controlling CRISPR-Cas9 genome editing in human cells using a molecular glue degrader
Source: Mol Ther Nucleic Acids. 2025 Jul 21;36(3):102640. doi: 10.1016/j.omtn.2025.102640 (PMC12341526; doi:10.1016/j.omtn.2025.102640)
Supplement: Document S1. Figures S1–S6, and Tables S1–S3 [file mmc1.pdf]

OMTN, Volume *36*

Supplemental information

Controlling CRISPR-Cas9 genome editing

in human cells using a molecular glue degrader

Namita Khajanchi, Vrusha Patel, Ronak Dua, Meha Kabra, Bikash R. Pattnaik, and Krishanu Saha

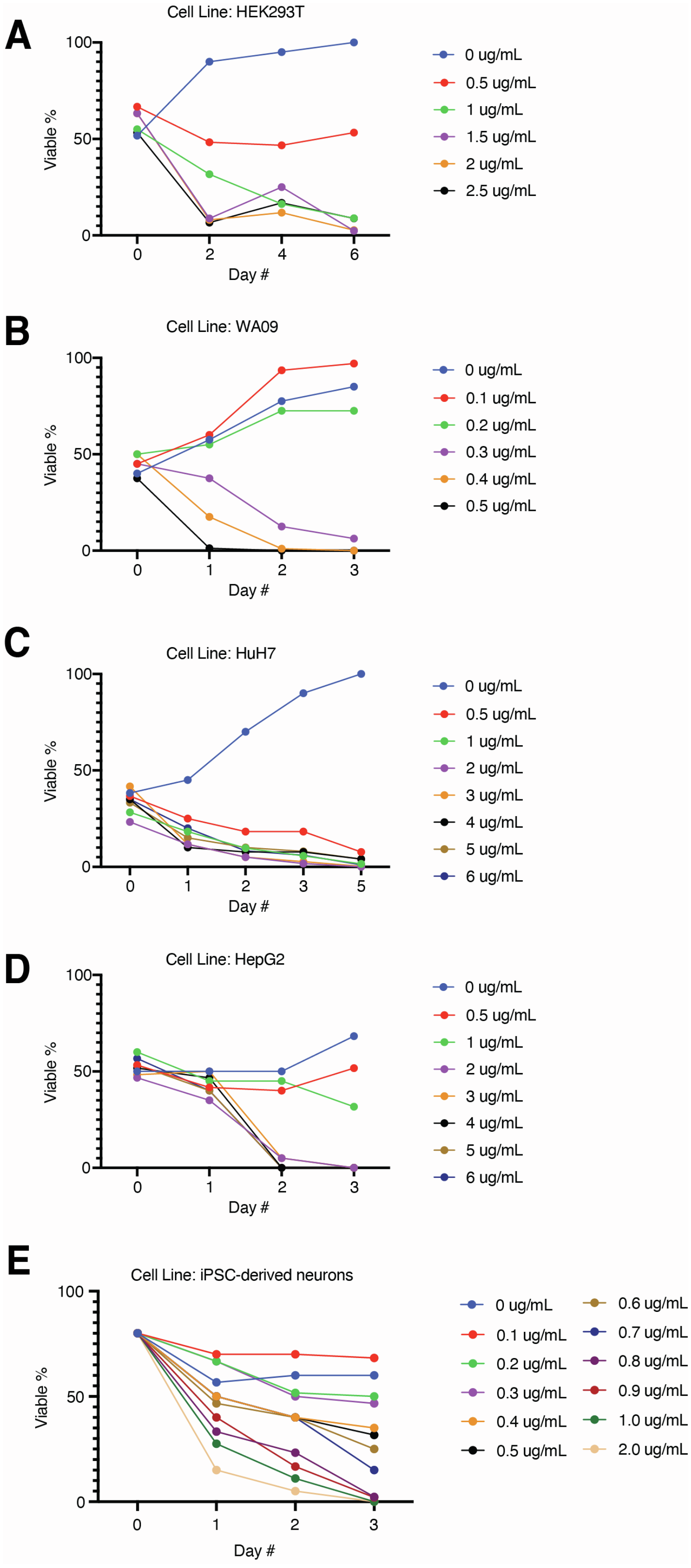
A
Cell Line: HEK293T
Viable %
Day #
0 ug/mL
0.5 ug/mL
1 ug/mL
1.5 ug/mL
2 ug/mL
2.5 ug/mL
B
Cell Line: WA09
Viable %
Day #
0 ug/mL
0.1 ug/mL
0.2 ug/mL
0.3 ug/mL
0.4 ug/mL
0.5 ug/mL
C
Cell Line: HuH7
Viable %
Day #
0 ug/mL
0.5 ug/mL
1 ug/mL
2 ug/mL
3 ug/mL
4 ug/mL
5 ug/mL
6 ug/mL
D
Cell Line: HepG2
Viable %
Day #
0 ug/mL
0.5 ug/mL
1 ug/mL
2 ug/mL
3 ug/mL
4 ug/mL
5 ug/mL
6 ug/mL
E
Cell Line: iPSC-derived neurons
Viable %
Day #
0 ug/mL
0.1 ug/mL
0.2 ug/mL
0.3 ug/mL
0.4 ug/mL
0.5 ug/mL
0.6 ug/mL
0.7 ug/mL
0.8 ug/mL
0.9 ug/mL
1.0 ug/mL
2.0 ug/mL

Figure S1. Puromycin (Puro) Kill Curves for **(A)** HEK293T, **(B)** WA09, **(C)** HuH7, **(D)** HepG2 cells, and **(E)** hiPSC-derived neurons. Cells were seeded at approximately 50% in all wells prior to starting the kill curve. **(A)** HEK cells were killed within 2 days with 1.5 - 2.5 μg/mL of puro. The lowest dose, 1.5 μg/mL puro was used. **(B)** WA09 were killed within a day with 0.5 μg/mL of puro and within 2 days with 0.4 μg/mL of puro. Because iPSCs are puromycin sensitive, 0.3 μg/mL puro was used. **(C)** HuH7 cells were killed within 5 days with 1 μg/mL - 6 μg/mL of puro. One of the lowest doses, 2 μg/mL puro was used to match the amount that is used in **(D)** HepG2 cells as both cells would be cultured at the same time. **(E) h**iPSC-neurons were killed within 3 days at puromycin concentrations of 0.8 μg/mL and higher. Since these neurons are sensitive, we used a lower dose of 0.7 μg/mL of puro. Data represented in line plots are means, n = 3 technical replicates per condition.

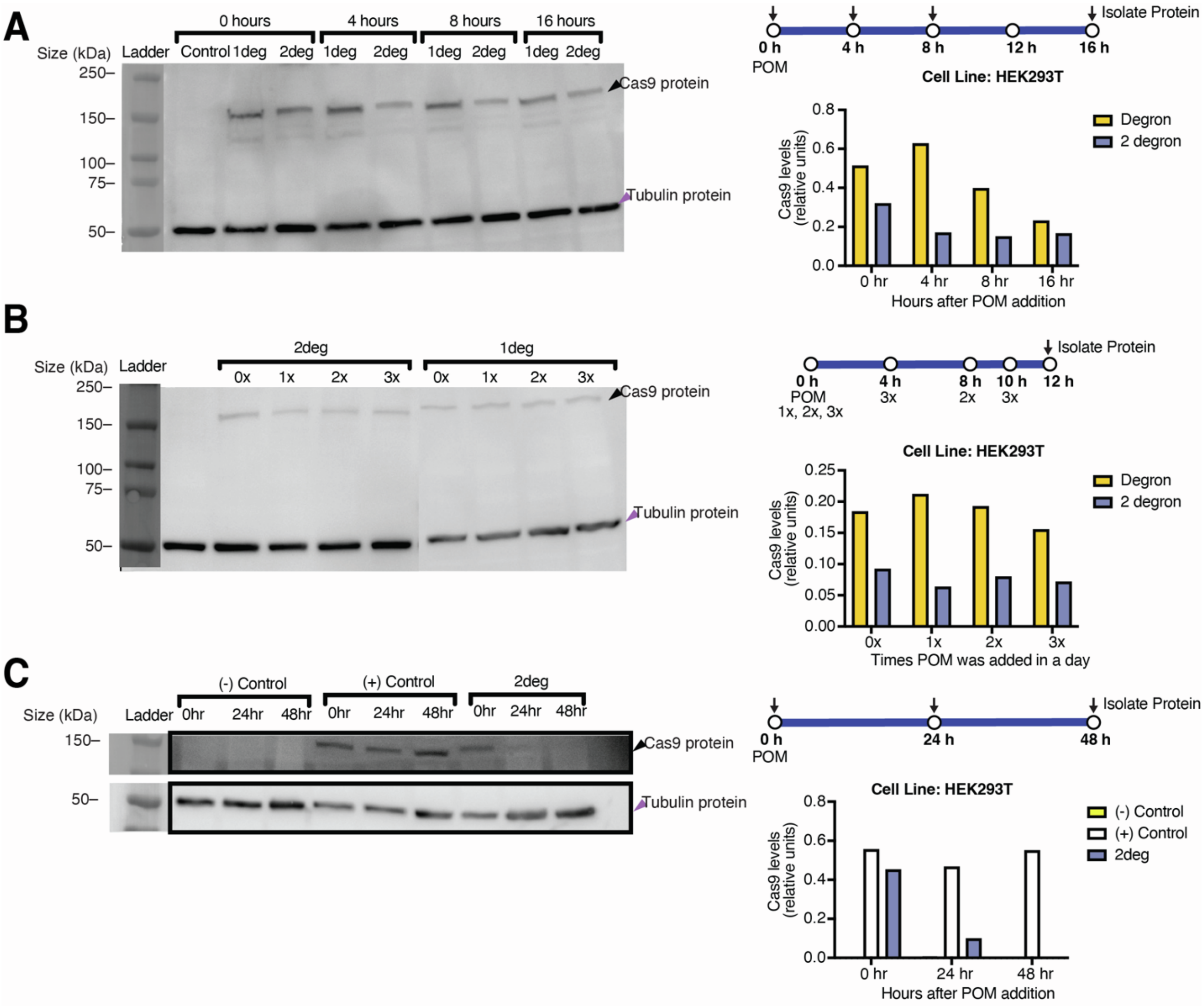

Figure S2. Western blots to determine degron construct to use. (**A**) Representative western blot for Cas9-1d and Cas9-d protein levels over the course of 16 hours after one dose of POM at hour 0. The half-life for Cas9-1d was 8.29 hours and for Cas9-d was 7.31 hours. Data represented in bar graphs are n = 1 sample. **(B)** Representative western blot showing Cas9 levels in cells after 24 hours when POM was added once, twice, or three times a day. There is no significant change in Cas9 levels. Data represented in bar graphs are n = 1 sample. (**C**) Representative western blot depicting untransduced cells (- Control), Cas9-mCherry (+ Control) and Cas9-d cells. POM was added to all cells at hour 0, and samples were isolated at 0, 24, and 48 hours. Data represented in bar graphs are n = 1 sample.

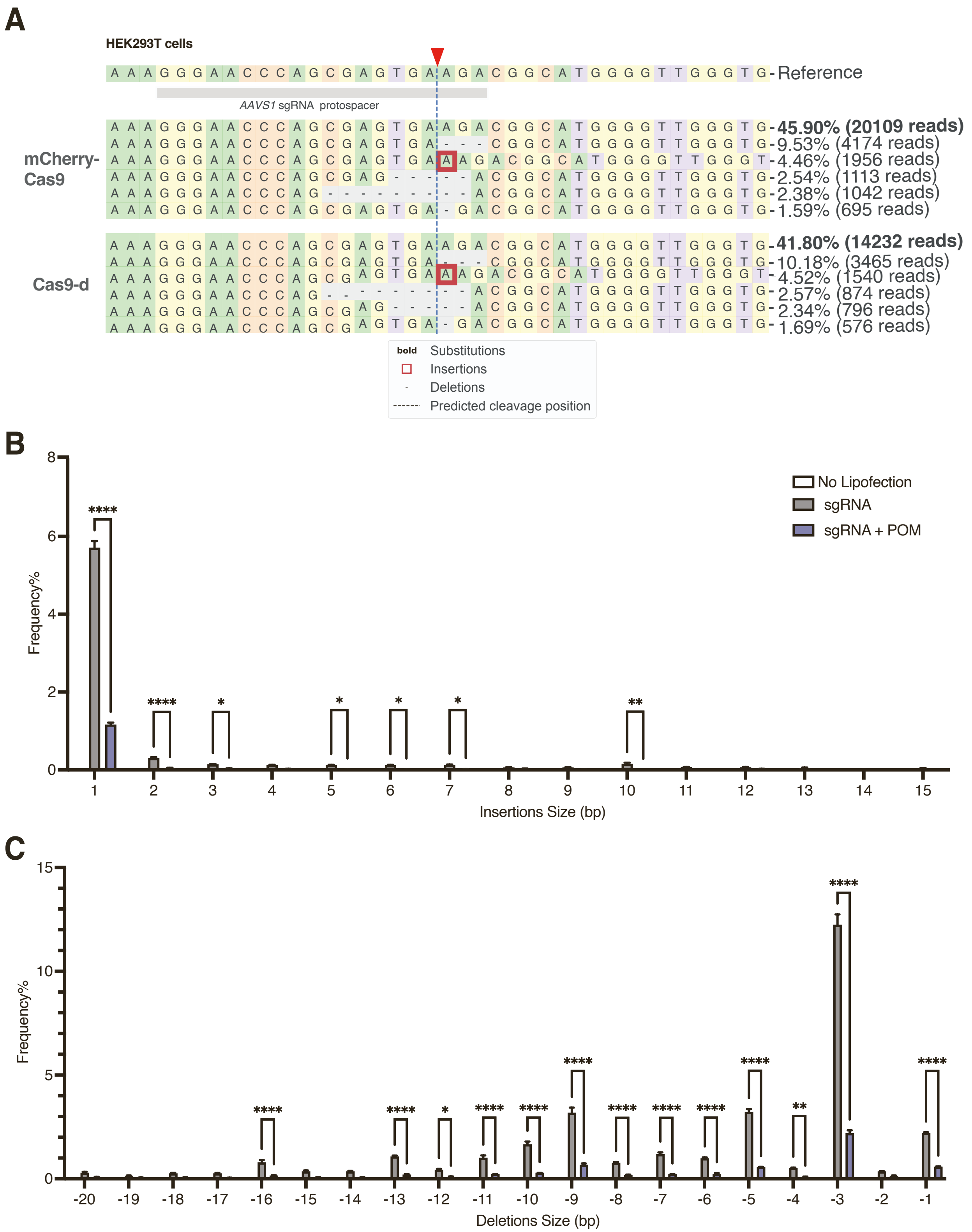

Figure S3. Decrease in indels in POM-induced Cas9-d HEK293T cells. Indel profiles after lipofection for **(A)** WT Cas9 (Cas9-mCherry) and Cas9-d at the *AAVS1* locus in HEK cells. The dotted line represents where the cut site is. Both indel profiles are similar except for extra inserts

in WT Cas9 cells. **(B)** The insertion spectrum at the AAVS1 on-target locus shows that there is an average of 4.9-fold change in frequency between significant sizes of insertions in POM-induced Cas9-d cells and Cas9-d cells. **(C)** The deletion spectrum at the AAVS1 on-target locus shows that there is an average of 9.1-fold change in frequency between significant sizes of deletions in POM-induced Cas9-d cells and Cas9-d cells. Data represented in bar graphs in (B) and (C) are represented as mean +/- SEM, n = 3 technical replicates per condition, p-values generated by two-way ANOVA; ns = $p \geq 0.05$ (not shown), * for $p < 0.05$, ** for $p < 0.01$, *** for $p < 0.001$, **** for $p < 0.0001$.

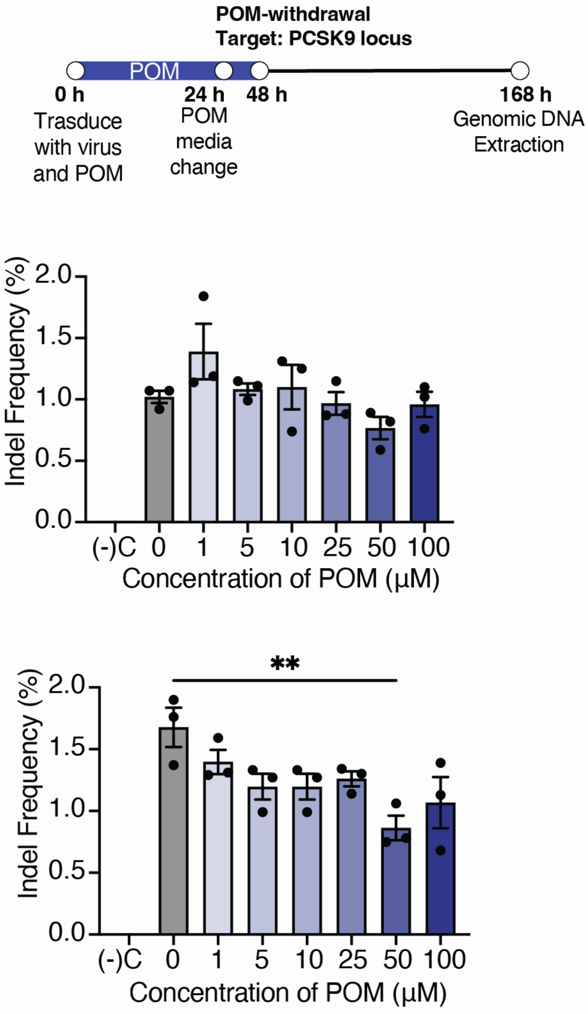

Figure S4. Hepatocyte withdrawal experiments.
On-target indel frequencies after 5 days of POM withdrawal within (top) HepG2 and (bottom) Huh7 cells. Editing increases upon POM at each concentration up to 2-fold when compared with POM-induced counterparts. Data represented in all bar graphs are represented as mean +/- SEM, n = 3 technical replicates per condition, p-values generated by two-way ANOVA; ns = $p \geq 0.05$ (not shown), * for $p < 0.05$, ** for $p < 0.01$, *** for $p < 0.001$, **** for $p < 0.0001$.

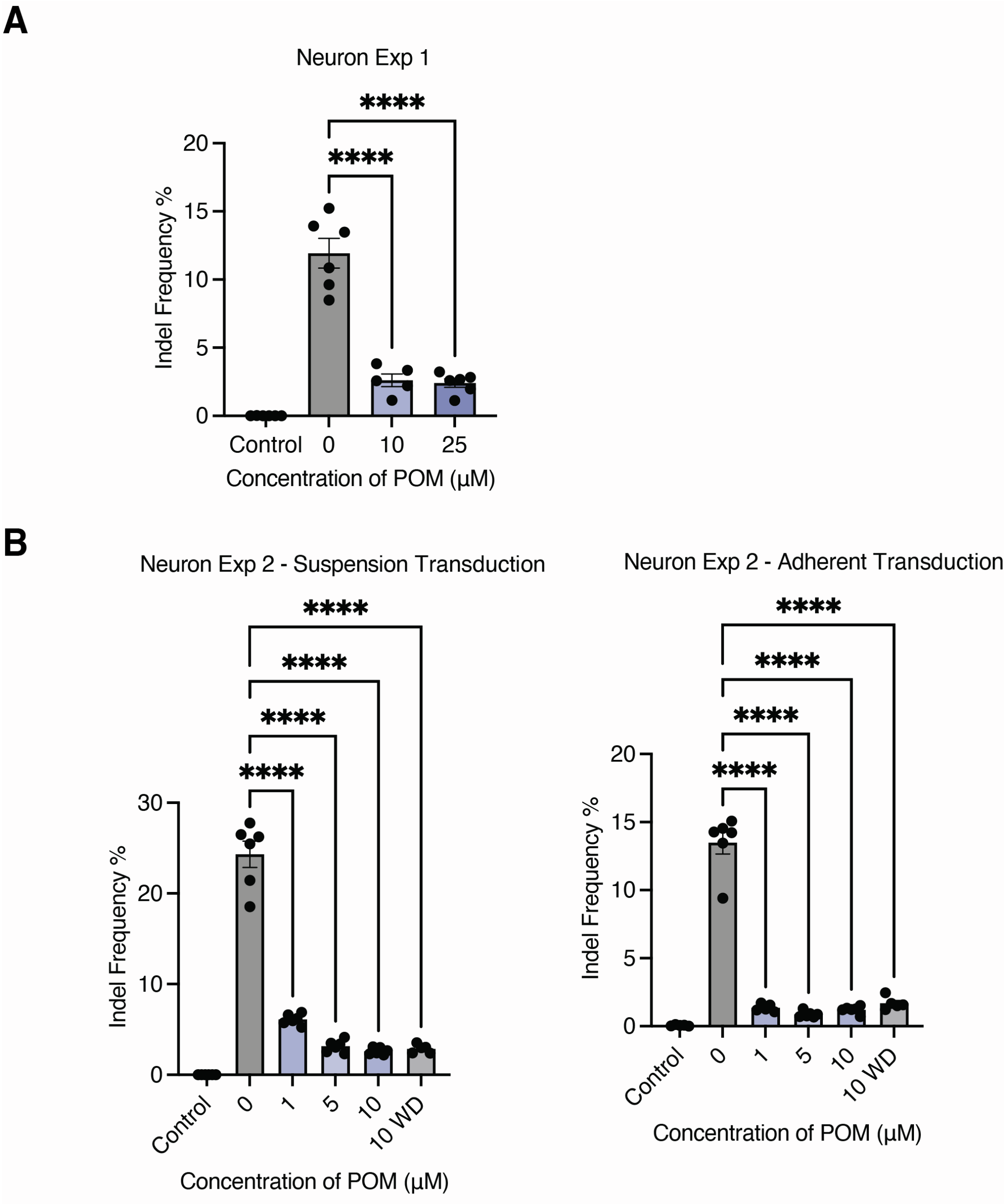

Figure S5. hiPSC-derived neuron replicates experiments.

(A) Neurons were transduced with Cas9-d virus while in suspension with POM concentrations of 0, 10, or 25 µM media on Day 0. On Day 1, there was a full media change with POM, followed by half a media change on Day 4 with POM, and genomic DNA extraction of Day 7. **(B)** Neurons were transduced either in suspension like **(A)** or while adherent. For neurons transduced while adherent, on Day 0, cells were seeded, on Day 1, cells were transduced with virus and POM. On Day 4, media was changed and on Day 7, genomic DNA was extracted. Data represented in bar graphs are represented as mean +/- SEM, n = 5 technical replicates per condition, p-values generated by two-way ANOVA; ns = $p \geq 0.05$ (not shown), * for $p < 0.05$, ** for $p < 0.01$, *** for $p < 0.001$, **** for $p < 0.0001$).

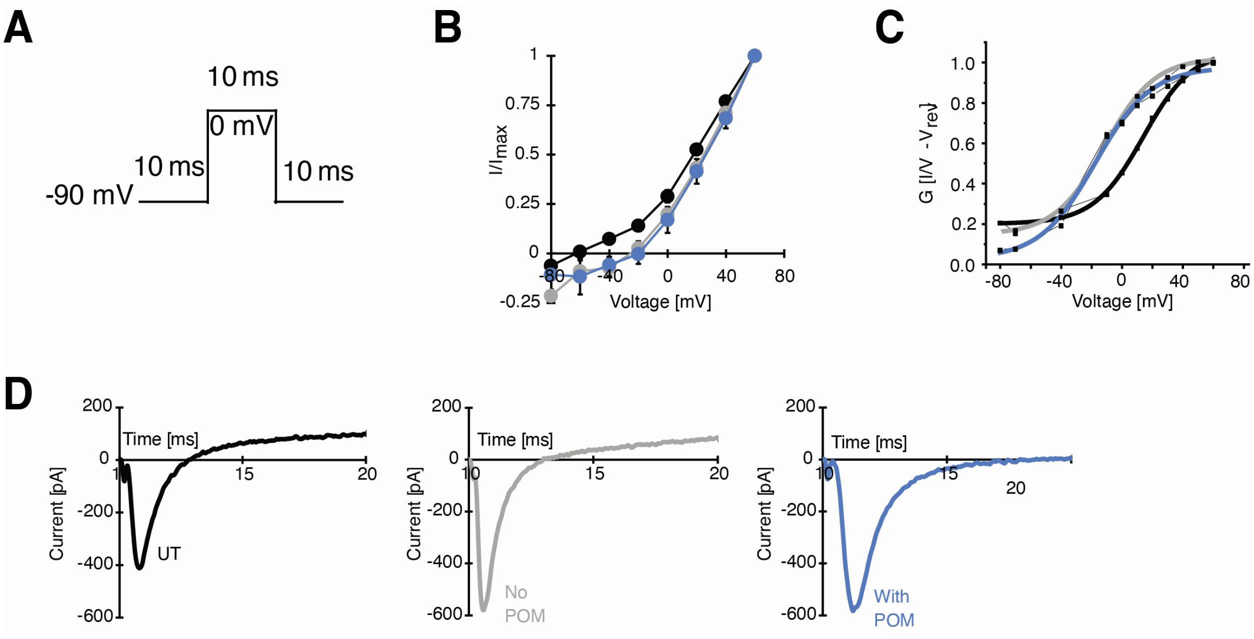

Figure S6. High-efficiency genome editing while preserving neuronal function in human hiPSC-derived cortical GABAergic neurons.
(A) Voltage response and Na current stimulation of a short (10 ms) hyperpolarizing current injection. **(B)** Normalized current (I/I_{max}), evoked by the same voltage steps (as shown in **Figure 5C**) in the neurons which were transduced with no POM (n=13), transduced with POM (n=12) and untreated (n=17). The error bar represents mean ± SE at each voltage. **(C)** Normalized conductance (G)-voltage plot in treated and untreated neurons. Solid line represents sigmoidal curve fit. **(D)** Na current in neurons, generated using a pulse protocol as shown in **A**. Untreated n = 17, No POM n = 13, POM n = 12.

Table S1. sgRNA targeting sequences.

	**sgRNA (5' - 3')**
AAVS1	GGGAACCCAGCGAGTGAAGA
VEGFA	GGGTGGGGGGAGTTTGCTCC
PCSK9	GGTGCTAGCCTTGCGTTCCG
APP	ATCCATTCATCATGGTGTGG

Table S2. PCR primers used for on- and off-target amplification of genomic loci for deep-sequencing.

	**Forward Primer Sequence (5' - 3')**	**Reverse Primer Sequence (5' - 3')**
AAVS1 On-Target	CTTCCTTCTCGGCGCTGCACCAC	AGCCAGGGAGACGGGGTACTTTGG
VEGFA On-Target	GCCCTGGGCTCTCTGTACAT	CACACCCCGGCTCTGGCTAA
PCSK9 On-Target	TGCTGCTGCTGCTCCTGGGT	GGAAGGTGGCTGTGGTTCCGTG
APP On-Target	GGTGTTGTCATAGCGACAGT	GCTAAGCCTAATTCTCTCA
VEGFA OTE1	ACTGCACCTGGCCATCATCC	TCTGTCCTATTTACACAGCAGACC
VEGFA OTE2	GTTCTGAGGCAGAGATGGCA	CATACAGGCCTGCTTCCTCC
VEGFA OTE3	TCCCCAGGGGACATTTGGCA	TGGGAGTTGTCCTGCAGCTGT

Table S3. Comparison of RNA expression of neo-substrates in different organs and cell types using Protein Atlas. The liver and kidney express high levels of the CRBN protein indicating it is sufficient for protein degradation to occur. Cell lines we used (HEK293T, Huh7, HepG2, inhibitory neurons) showed high levels of RNA expression for the CK1α and GSPT1 genes when compared to other genes. Data was not available for ARID2, SALL4, RNF166, and ZNF692 and protein expression data was not available for the eye (retina). (nTPM is transcripts per million)

Protein	What does it do?	Organ	Protein Expression	nTPM	Cell Type	nTPM	Reference
CRBN Cereblon	Plays a role in cellular and tissue metabolism, influences regulation of ion transport and AMP-activates protein kinase (AMPK) signaling pathway.	Cerebral cortex (brain) - neuronal cells	Medium	31.9	Inhibitory Neurons	70.9	Shi Q, Chen L, 2017
		Kidney	High	27.4	HEK293T	26.7	
		Liver - Hepatocytes	High	33.1	HepG2	16.6	
					Huh7	14.9	
		Eye (Retina)	No Data	21	hTERT-RPE1	32.8	
GSPT1 G1 to S phase transition 1	Translation termination factor crucial for releasing newly synthesized polypeptides from the ribosome by interacting with eukaryotic translation termination factor 1 (ETF1)	Cerebral cortex (brain) - neuronal cells	Medium	34.9	Inhibitory Neurons	45.1	NCBI Gene 2935
		Kidney	High	51.8	HEK293T	144.2	
		Liver - Hepatocytes	Medium	74	HepG2	281.3	
					Huh7	111.1	
		Eye (Retina)	No Data	34.2	hTERT-RPE1	118.1	
CSNK1A1 Casein kinase 1 alpha 1	Regulates cellular processes and signaling pathways	Cerebral cortex (brain) - neuronal cells	Not Detected	53.7	Inhibitory Neurons	110.5	Jiang S, et al., 2018
		Kidney	Low	48.5	HEK293T	73.2	
		Liver - Hepatocytes	Not detected	66.1	HepG2	110	
					Huh7	102.6	
		Eye (Retina)	No Data	55.9	hTERT-RPE1	158.1	
ZFP91 Zinc finger protein, atypical E3 ubiquitin ligase	Regulates the non-canonical NF-κB signaling pathway.	Cerebral cortex (brain) - neuronal cells	High	23.6	Inhibitory Neurons	0	Sun SC, 2010
		Kidney	High	24.2	HEK293T	22.8	
		Liver - Hepatocytes	High	29.7	HepG2	27	
					Huh7	23.8	
		Eye (Retina)	No Data	28.9	hTERT-RPE1	47.6	
	Terminates protein synthesis when interacting	Cerebral cortex (brain) - neuronal cells	Medium	10	Inhibitory Neurons	4.2	Betram G, et al. 2000

GSPT2 G1 to S phase transition 2	with eukaryotic release factor 1 (eRF1)	Kidney	Medium	6.1	HEK293T	11.9	
		Liver - Hepatocytes	Low	10.9	HepG2	0	
					Huh7	0	
		Eye (Retina)	No Data	7.5	hTERT-RPE1	13.1	
ARID2 AT-rich interaction domain 2	Regulates gene expression, chromatin organization, and cell differentiation.	Cerebral cortex (brain) - neuronal cells	No Data	3.8	Inhibitory Neurons	57.8	Bluemn T, et al., 2021
		Kidney	No Data	6.1	HEK293T	9.2	
		Liver - Hepatocytes	No Data	5.2	HepG2	10.2	
					Huh7	12	
		Eye (Retina)	No Data	12.8	hTERT-RPE1	12.9	
SALL4 Spalt like transcription factor 4	Transcription factor for maintaining stem cell pluripotency and self-renewal	Cerebral cortex (brain) - neuronal cells	No Data	0.1	Inhibitory Neurons	3.4	Tatestsu H, et al. 2016
		Kidney	No Data	0.8	HEK293T	0	
		Liver - Hepatocytes	No Data	1.5	HepG2	0.6	
					Huh7	1.8	
		Eye (Retina)	No Data	0.5	hTERT-RPE1	0	
RNF166 Ring finger protein 166	Mediates both Lys63-linked ubiquitination and sumoylation of its target proteins	Cerebral cortex (brain) - neuronal cells	No Data	18.7	Inhibitory Neurons	4.6	Hwang IY, et al., 2021
		Kidney	No Data	20.1	HEK293T	20.4	
		Liver - Hepatocytes	No Data	22.8	HepG2	23.4	
					Huh7	15.9	
		Eye (Retina)	No Data	3.3	hTERT-RPE1	6.6	
ZNF692 Zinc finger protein 692	Influences the shape of the nucleolus by interacting with nucleophosmin 1 (NPM1) and changing how it assembles itself	Cerebral cortex (brain) - neuronal cells	No Data	30.5	Inhibitory Neurons	18.9	Brown IN, et al., 2024
		Kidney	No Data	26.3	HEK293T	5.3	
		Liver - Hepatocytes	No Data	21.2	HepG2	4.4	
					Huh7	6.2	
		Eye (Retina)	No Data	8.2	hTERT-RPE1	5.6	
