# Supplementary material for: Controlling CRISPR-Cas9 genome editing in human cells using a molecular glue degrader
Source: Mol Ther Nucleic Acids. 2025 Jul 21;36(3):102640. doi: 10.1016/j.omtn.2025.102640 (PMC12341526; doi:10.1016/j.omtn.2025.102640)
Supplement: Document S2. Article plus supplemental information [file mmc2.pdf]

Molecular Therapy
Nucleic Acids
Original Article

Controlling CRISPR-Cas9 genome editing in human cells using a molecular glue degrader

Namita Khajanchi,[1,2] Vrusha Patel,[1,3] Ronak Dua,[1,4] Meha Kabra,[5,6] Bikash R. Pattnaik,[5,6,7] and Krishanu Saha[1,2,5,6,8]

[1]Department of Biomedical Engineering, University of Wisconsin-Madison, Madison, WI 53706, USA; [2]Wisconsin Institute for Discovery, University of Wisconsin-Madison, Madison, WI 53715, USA; [3]Department of Integrative Biology, University of Wisconsin-Madison, Madison, WI 53706, USA; [4]Department of Bacteriology, University of Wisconsin-Madison, Madison, WI 53706, USA; [5]Department of Pediatrics, University of Wisconsin-Madison, Madison, WI 53792, USA; [6]McPherson Eye Research Institute, University of Wisconsin-Madison, Madison, WI 53705, USA; [7]Department of Ophthalmology and Visual Sciences, University of Wisconsin-Madison, Madison, WI 53705, USA; [8]Center for Human Genomics and Precision Medicine, University of Wisconsin-Madison, Madison, WI 53705, USA

CRISPR-Cas9-based genome editors can precisely target and edit genes efficiently. However, prolonged Cas9 activity poses challenges for laboratory experiments and raises safety concerns for therapeutic applications due to unintended consequences such as off-target editing, genotoxicity, immunogenicity, and undesired on-target modifications. Here, we evaluate a novel molecular glue degradation system, called Cas9-degron (Cas9-d), designed to degrade Cas9 in the presence of the US Food and Drug Administration (FDA)-approved drug, pomalidomide (POM). This system is highly biocompatible and rapidly reduces Cas9 protein levels within 4 h of induction, resulting in a 3- to 5-fold decrease in editing at on-target sites. The reduction is reversible, as Cas9 levels are restored within 24 h after POM withdrawal. Without initiating degradation, the on-target editing efficiency and accuracy of the Cas9-d system remain intact in different human cell types, including hepatic cell lines and human induced pluripotent stem cell (hiPSC)-derived GABAergic neurons. Cells edited with the Cas9-d system were healthy and functional, exhibiting minimal toxicity from using the strategy. The Cas9-d system provides a versatile approach to adjust Cas9 levels, demonstrating its potential as an experimental tool for controlling genome editing outcomes *in vitro* and *ex vivo*. With further development, it holds promise for enhancing somatic cell genome editing *in vivo*.

INTRODUCTION

CRISPR-based genome editors are powerful tools that can edit the genome in many cells across biology, including human somatic cells in patients.[1] While transient delivery of CRISPR base editors can be safely dosed into infants,[2] extended constitutive expression of editor proteins after the delivery of nucleic acids encoding the CRISPR system can result in unwanted outcomes, such as unintended genomic modifications to the target site (i.e., large deletions and chromothripsis),[3–9] off-target editing,[10,11] and toxicity to cells.[12,13] Prolonged exposure to Cas9 increases adverse effects, including off-target editing and undesired on-target modifications such as mono-allelic deletions or loss of heterozygosity.[14] *Ex vivo* primary cell applications also suffer from prolonged Cas9 exposure, as CRISPR-Cas9 genome editing can induce chromosome loss in human primary T cells.[15,16] Additionally, engineered CAR-T cells for clinical use and general CRISPR gene editing have been associated with cell toxicity and genomic instability mediated potentially by altered tumor suppressor pathways. These outcomes are key concerns for regulatory agencies, especially when targeting organs other than the liver *in vivo*, where high levels of editor proteins may be required for efficient gene editing to occur.

Apart from therapeutic applications *in vivo*, prolonged Cas9 activity presents significant challenges across diverse laboratory applications. Undesired on-target outcomes have been observed in CRISPR screening applications using experimental tools, resulting in artifacts.[17] In embryonic development, prolonged Cas9 activity leads to heterogeneous genome-editing outcomes and mosaicism in embryonic editing, while also inducing genotoxicity and immunogenicity that can compromise developmental processes.[18] Virus-mediated expression of CRISPR-Cas9 is commonly used for genome editing in animal brains to model or treat neurological diseases, but the potential neurotoxicity of overexpressing bacterial Cas9 in the mammalian brain remains unknown. However, studies suggest that reducing Cas9 half-life can mitigate these effects.[19] In microbial systems, consistently high levels of Cas9 expression are thought to be harmful to various microbial species. This is demonstrated by the significant decrease in transformants, with proposed mechanisms including off-target binding of Cas9 to bacterial gene promoters, which results in transcriptional silencing and cellular toxicity.[20,21] These different toxicity mechanisms emphasize the critical importance of temporal control over Cas9 expression to minimize cellular toxicity while preserving editing efficiency in numerous laboratory settings.

Control strategies for CRISPR systems can be divided into three biological regulation mechanism categories: activation (i.e., "on"

Received 16 February 2025; accepted 16 July 2025;
https://doi.org/10.1016/j.omtn.2025.102640.

Correspondence: Krishanu Saha, Center for Human Genomics and Precision Medicine, University of Wisconsin-Madison, Madison, WI 53705, USA.
E-mail: ksaha@wisc.edu

Published by Elsevier Inc. on behalf of The American Society of Gene and Cell Therapy.
This is an open access article under the CC BY license (http://creativecommons.org/licenses/by/4.0/).

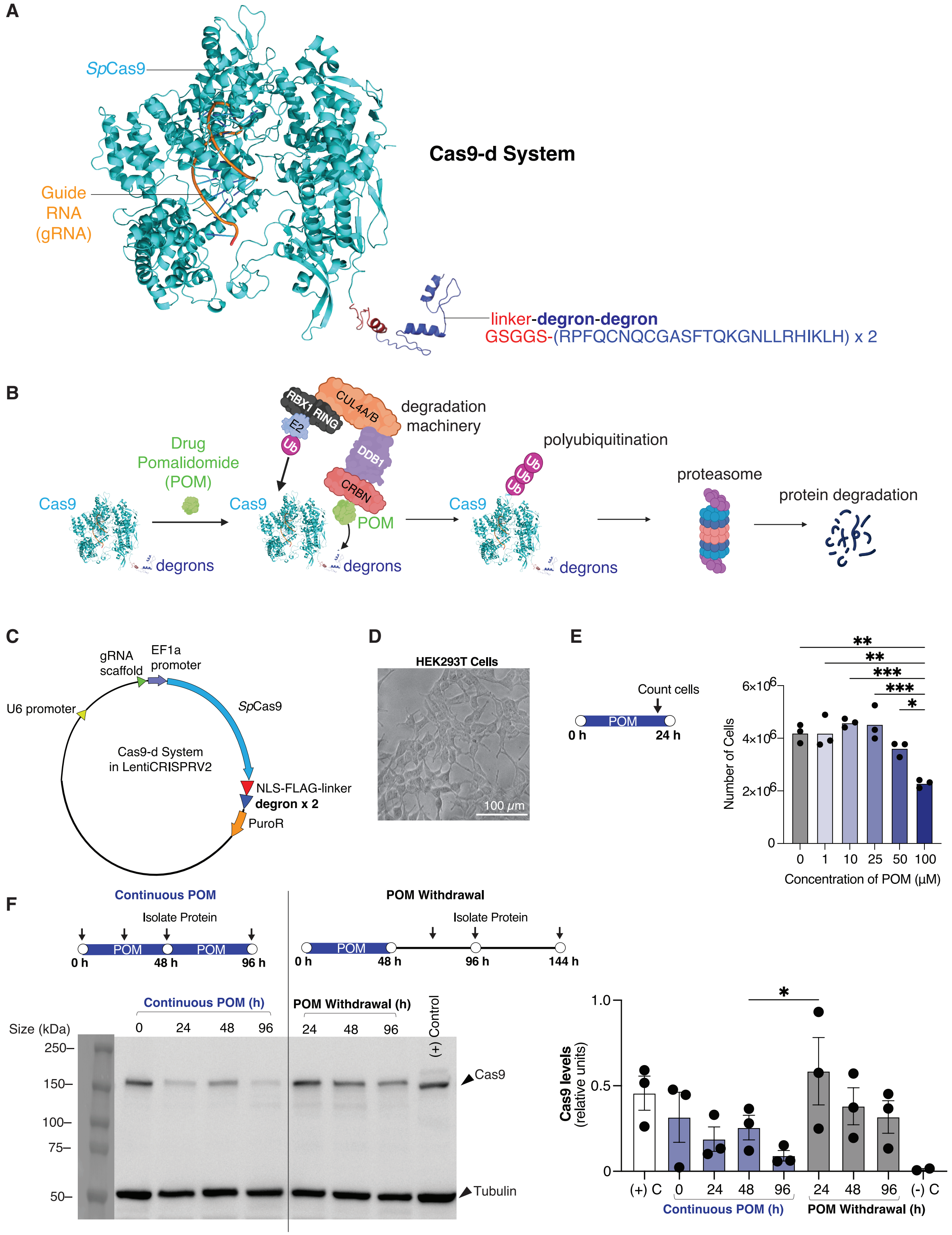

(legend on next page)

switches), inhibition (i.e., "off-switches"), and degradation (i.e., "off-switches"). First, for activation, small-molecule inducible domains can be engineered to stabilize and activate Cas9,[22–24] but they have several limitations. Systems using doxycycline often have leaky activity[25] and require several days to reach peak Cas9 activity.[24,26,27] This prolonged activation period could increase adverse events from prolonged nuclease activity in the cell. To gain more refined control of the kinetics of genome editing, small molecules can drive the fusion of the split N- and C-terminal fragments of Cas9, leading to the formation of active Cas9.[28–30] One such split system relies on the interaction between the FK506-binding proteins (FKBP) and FKBP-rapamycin binding (FRB) domains. In the presence of rapamycin, which can cross the blood-brain barrier,[31] these domains dimerize and reconstitute active Cas9, enabling genome editing. In these split systems, each portion of Cas9 has its nuclear localization signal (NLS); if only one terminal fragment enters the nucleus or if non-covalent protein dimerization occurs, Cas9 will fail to reconstitute fully and be functionally inactive. Another variation of the split system involves linking each half of Cas9 with the ligand-binding domain of the estrogen receptor (ERT), allowing control of Cas9 expression and nuclear translocation via the synthetic ligand 4-hydroxytamoxifen (4-OHT).[26,32] This strategy yields rapid activation and high specificity, with lower background activity compared to wild-type (WT) Cas9. Moreover, these split Cas9 systems can be delivered in two separate vectors, offering a solution to the vector packaging limitations faced in traditional genome editing approaches.[33–36] Even with high delivery efficiencies and deep penetration of small molecules into tissues, obtaining high activity upon reconstitution of the full Cas9 and complete deactivation upon washout can be challenging *in vivo*. However, some of the strategies, such as the FKBP-FRB system, mentioned previously, are irreversible upon activation. Once Cas9 forms, it is constitutively active, and this prolonged expression of Cas9 can lead to unintended modifications in the genome.

For inhibition control strategies, anti-CRISPR proteins can effectively and reversibly inhibit or deactivate functional Cas9, serving as "off-switches" for Cas9 activity.[37,38] One class of anti-CRISPR proteins inhibits Cas9 interaction with the protospacer adjacent motif (PAM) site, while another binds to Cas9's endonuclease domain to block DNA cleavage.[37,39,40]

Additional methods to further reduce nuclease levels after successful editing could enhance the biocompatibility and precision of using CRISPR-based nucleases. Degradation strategies for completely removing Cas9 may eliminate undesirable activity from Cas9 within the cell. In mammalian cells, the ubiquitin-proteasome pathway plays a key role in the degradation of most proteins.[41] This pathway targets degrons, i.e., degradation signals, on the protein. Degrons are typically short peptide sequences that flag the protein for degradation. These sequences are recognized by three cooperating specific types of enzymes—E1, E2, and E3. The E3 enzyme, known as the ubiquitin ligase, recognizes the degrons on target proteins and facilitates the transfer of ubiquitin from the E2 enzyme to the target protein.[42] Once the target protein is tagged with ubiquitin molecules, it is marked for destruction and degraded by the proteasome. Several degron systems include the auxin-induced degradation (AID) system,[43–45] degradation tag (dTag)[46] system, and the small molecule-assisted shutoff (SMASh) system.[47]

Using a degron derived from the lymphoid-restricted transcription factor IKZF3,[48] we created a Cas9-degron (Cas9-d) system that can be targeted for degradation with a small molecule, pomalidomide (POM). The US Food and Drug Administration (FDA) and other regulatory agencies have approved POM for the treatment of multiple myeloma. It binds to cereblon (CRBN),[49] a component of the ubiquitin E3 ligase complex, and promotes the recruitment of IKZF3 to the E3 complex, leading to substrate (Cas9-d) ubiquitination and degradation, as shown in Figure 1A.[50–52] Unlike the AID and dTAG system, the IKZF3 degron in Cas9-d is short, composed of only 25 amino acids, making it more efficient to insert into delivery vectors such as adeno-associated viruses (AAV),[53,54] nonviral lipid nanoparticles (LNPs),[55–57] and nonviral nanocapsules,[58,59] which have limited payloads. Editing efficiency and specificity of Cas9-d were assessed in several human cells, including hepatic and neuronal cell types. This system rapidly decreases Cas9 levels within hours of POM induction, is reversible, and preserves the on-target editing efficiency and precision of the Cas9 nuclease. The development of the Cas9-d system can facilitate the characterization of kinetics and safety profiles of genome editors, helping to mitigate unintended and adverse effects associated with constitutively active Cas9 editors.

Figure 1. Cas9-d system controls nuclease levels within human cells in a reversible manner dependent on pomalidomide

(A) Schematic and amino acid sequence for the Cas9-d system: *Sp*Cas9, a nuclear localization signal (NLS), a FLAG tag, and a linker to fuse the two degrons to Cas9. The model structure shows *Sp*Cas9 in cyan, the double degron in blue, and the NLS-FLAG-linker in red. This model displays the relative difference in size among the Cas9, degrons, tags, and linkers. (B) Schematic of pomalidomide (POM) binding to the Cas9-degron system to target it to the proteosome system. Adding POM to Cas9-degron initiates the cullin-RING E3 ubiquitin ligase 4-cereblon (CRL4-CRBN) complex, which ubiquitinates Cas9 and degrades the nuclease via the proteasome. (C) LentiCRISPR V2 Cas9-d construct used for HEK293T and WA09 cells including a cytomegalovirus (CMV) promoter, U6 promoter controlling the transcription of the gRNA, and EF1α promoter controlling the transcription of the wild-type Cas9 with degron as well as the puromycin resistant (PuroR) gene. (D) Representative image of edited HEK293T cells. Scale bar, 100 μm. (E) Live HEK293T cells with various POM concentrations. Live and dead cells were distinguished using 0.4% trypan blue. Cells treated with POM concentrations of 0–50 μM exhibited minimal cell death. Data represented in bar graphs are represented as mean ± SEM, $n = 3$ technical replicates per condition, p values generated by one-way ANOVA comparison to 0 μM POM; ns = $p \geq 0.05$ (not shown), *$p < 0.05$, **$p < 0.01$, ***$p < 0.001$, and ****$p < 0.0001$. (F) Representative western blot for Cas9 protein levels upon addition and withdrawal (W/D) of POM. POM was added to cells at 0 h, and samples were isolated for western blotting at 24, 48, and 96 h. POM was also washed from cells (POM withdrawal, W/D) 48 h after incubation via PBS. Within 24 h, nuclease levels increased significantly over 2-fold. On right, quantification of nuclease levels including additional replicates. Data represented in bar graphs are represented as mean ± SEM, $n = 3$ technical replicates per condition, p values generated by two-way ANOVA multiple comparisons test between all samples except for the positive and negative controls; ns = $p \geq 0.05$ (not shown), *$p < 0.05$ and **$p < 0.01$.

RESULTS

Cas9-d design

We fused canonical *Sp*Cas9[60] (WT Cas9) to a degron sequence derived from IKZF3[48] POM can then bind to the ubiquitin E3 ligase complex and promote the recruitment of IKZF3-degron to the E3 complex, leading to substrate ubiquitination and degradation (Figures 1A and 1B).[50–52] The degron sequence is taken from the IKZF3 zinc finger 2 amino acids 144–168. This 25mer degron amino sequence is RPFQCNQCGASFTQKGNLLRHIKLH (Figure 1A). The Cas9 degron system is constructed using a WT Cas9, an NLS, a FLAG tag, and a linker (GSGGS) with one or two degron(s). This Cas9-d sequence was incorporated into a lentiviral vector for delivery into human cells, driven by a constitutive EF-1α core promoter, along with a downstream puromycin resistance gene (PuroR) to facilitate selection of transduced cells (Figures 1A and 1C). Lentiviral vectors have been used in several clinical trials,[61–64] and therefore provide a model for future *in vivo* gene therapy delivery applications.

To test the effects of the number of degrons on our Cas9, we expressed either one or two degrons in our Cas9-degron systems in HEK293T cells. Our one degron system (Cas9-1d) contains a single *linker*-degron (*GSGGS*-RPFQCNQCGASFTQKGNLLRHIKLH) fused to Cas9 at the C-terminus, while the two degron system (Cas9-d) is made up of 58 amino acids: *linker*-degron-*truncated-linker*-degron (*GSGGS*-RPFQCNQCGASFTQKGNLLRHIKLH-*GGS*-RPFQCNQCGASFTQKGNLLRHIKLH). An all-in-one vector encoded the Cas9-d system and the single guide RNA (sgRNA) in several constructs. In these vectors, after digesting the vector with the *BsmBI* restriction enzyme, a set of annealed oligonucleotides containing the sequence for sgRNA and PAM (designed to target specific genes) was incorporated into the sgRNA scaffold. The sgRNA, integrated into the scaffold, is under the control of the U6 promoter. After generating lentiviral particles and cell transduction, cells were subjected to antibiotic selection with puromycin, leading to the survival and proliferation of only those cells in which the Cas9-d vector sequences had been successfully integrated. A kill curve was generated for each cell line (Figure S1). The optimal concentration of puromycin for robust selection was 1.5 μg/mL for HEK293T cells, and 0.3 μg/mL for WA09 human pluripotent stem cells (hPSCs), 2 μg/mL for Huh7 and HepG2 cells, and 0.7 μg/mL for hiPSC-derived neurons in later experiments. Following the successful integration of the construct into HEK293T cells (Figure 1D), varying concentrations of POM were titrated over 24 h to maximize Cas9 degradation while minimizing cellular stress. Cells treated with POM concentrations of 0, 1, 10, 25, and 50 μM exhibited minimal cell death, while cells treated with 100 μM exhibited higher cell death and up to 45% toxicity compared to cells without POM (Figure 1E). Cells cultured with 1–50 μM POM thrived, while cells with 100 μM POM had significantly slower growth rates. An intermediate dose at 25 μM POM did not significantly affect cell growth or induce cell death and, therefore, was used for subsequent experiments.

We monitored nuclease protein levels at various times after treatment with POM. First, we tested Cas9 protein levels via western blotting for the Cas9-1d and Cas9-d constructs in HEK293T cells over 16 h after a single dose of POM at hour 0. Post-POM, Cas9-1d exhibited a 54% level reduction after 16 h, while Cas9-d exhibited a 53% level reduction after 8 h (Figure S2A). The half-life of Cas9-1d was calculated to be 8.29 h, while the half-life of Cas9-d is 7.31 h when accounting for changes between 4 and 16 h of Cas9-1d and 0 and 8 h for Cas9-d. In the plasma of multiple myeloma patients, the average half-life of POM is approximately 7.5 h. In the plasma of healthy subjects, the average half-life of POM is approximately 9.5 h.[65] Given this relatively short duration, we attempted to administer POM multiple times throughout the day to evaluate whether repeated dosing could further reduce Cas9 protein levels. There was a negligible difference in whether POM was added once, twice, or three times a day for both Cas9-1d and Cas9-d (Figure S2B). Since the Cas9-d (with two degrons) had a shorter half-life and degraded more rapidly, it was used in subsequent experiments. Extending the observation period to 48 h revealed a complete absence of Cas9-d protein in one western blot (Figure S2C). Upon further experimentation with more replicates, we observed that after 96 h on POM, Cas9-d protein levels decreased on average by 71% (Figure 1F).

Reversibility of Cas9-d

To explore the possibility of multiple rounds of nuclease activity, we investigated the increase of Cas9 levels upon withdrawal of POM from the HEK293T cells. Cells harboring a lentiCRISPRv2 cassette with *Sp*Cas9 without a degron but fused to a 2A-mCherry served as the positive control (WT Cas9), while untransduced cells served as the negative control. Using the observed stability of the Cas9-d decrease in cells after 16–24 h from Figure S2A, we extended the data collection interval from 4 h to 24 h and continued sampling every 24 h thereafter. Cells with Cas9-d were treated with 25 μM POM and collected for western blotting after 24, 48, and 96 h. A single dose of POM continuously degraded Cas9 protein for up to 96 h (Figure 1F). For POM withdrawal experiments, cells were exposed to POM for 48 h, and then POM was washed out with phosphate-buffered saline. Cas9-d protein levels rose 129% within 24 h of POM withdrawal (Figure 1F).

Cas9-d maintains on-target editing efficiency

To determine whether Cas9-d nuclease activity is preserved, we transfected a sgRNA into the HEK293T cells containing the Cas9-d system without an integrated sgRNA cassette. A previously characterized sgRNA against the *AAVS1* genetic locus[66] (Figure 2A, sgRNA sequence against site 10 is included in Table S1) was encapsulated in lipofectamine particles for delivery into HEK293T cells. WT Cas9 and untransduced cells were used as control conditions. One hour prior to lipofection, cells were treated with 25 μM POM in fresh media (Figure 2B). The sgRNA was encapsulated and then added to the cells. The cells were left undisturbed for 48 h after lipofection. Following this incubation period, genomic DNA was extracted for further analysis. Subsequent PCR amplification was performed around the targeted *AAVS1* locus in the genome (primers included

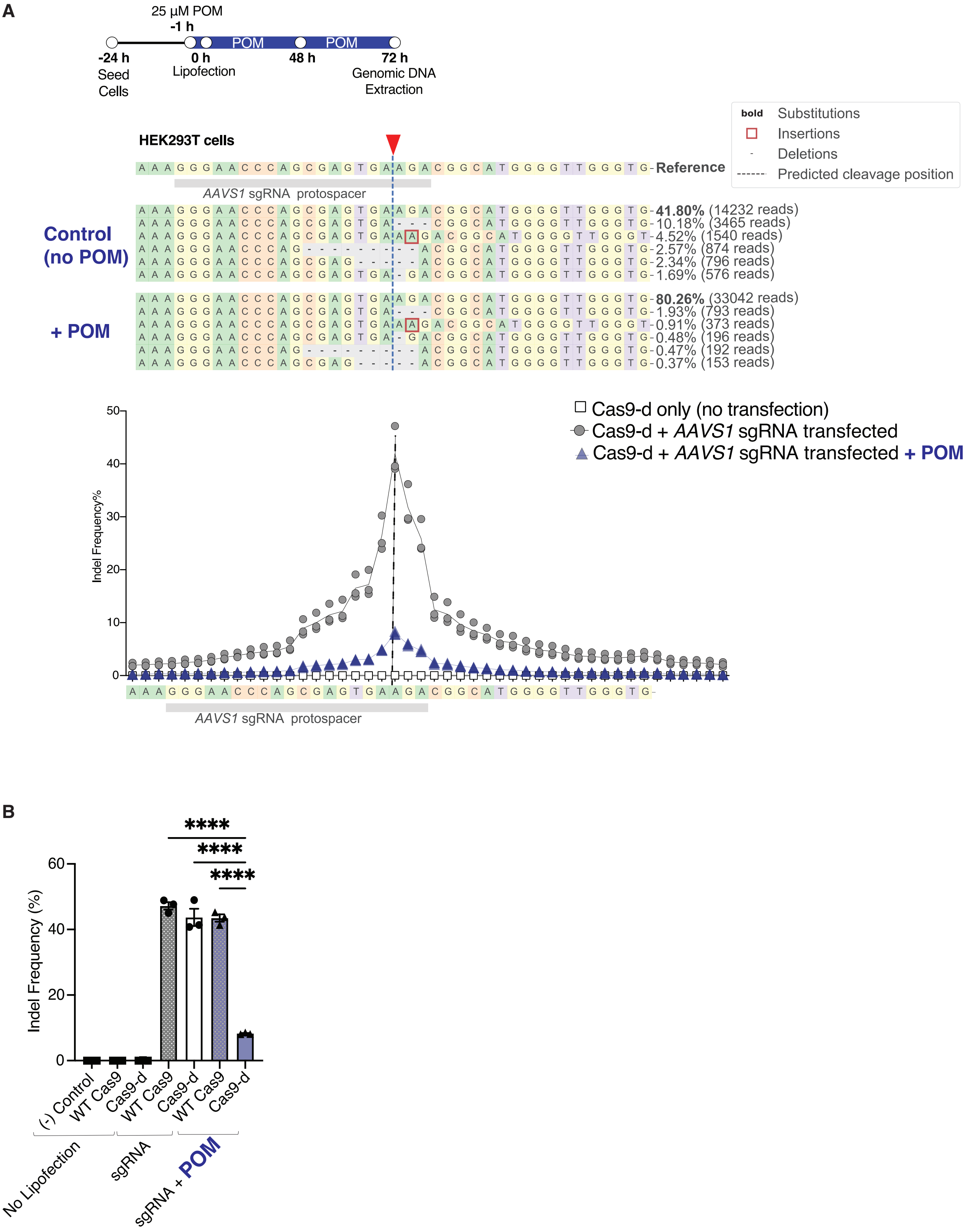

(legend on next page)

in Table S2), subjected to next-generation sequencing (NGS), and analyzed in the CRISPResso2 software.

On-target editing efficiency, as measured by indel frequency percentage at the on-target *AAVS1* site, was 43.7 ± 2.63% for the Cas9-d system, compared to 47.2 ± 1.13% for WT Cas9, confirming that the degron units do not hinder Cas9 activity (Figure 2B). Upon addition of POM, editing efficiency decreased by 5-fold from 43.7 ± 2.63% to 8.27 ± 0.159% (Figure 2B). The indel profiles of WT Cas9 and Cas9-d remain consistent in the absence of POM (Figure S3A). However, upon adding POM, the indel spectrum associated with Cas9-d shifted toward fewer and shorter deletions (Figure 2A). The indel profile at this site is detailed in Figure S3 with a 1-base pair (bp) insertion (Figure S3B) and a 3-bp deletion (Figure S3C) being most common. These data led us to evaluate whether Cas9-d cells with POM will also have lower off-target editing activity when compared to Cas9-d cells without POM.

Assessing off-target events after inducing degradation

While HEK293T cells have high transfection efficiency, their hypotriploid nature[67] and chromosomal instability[68,69] may not accurately mimic the biology of euploid cells in a patient. In contrast, hPSCs are genomically stable euploid cells useful for modeling, drug discovery and screening, cell therapy, and tissue engineering.[70–72] Hence, we evaluated on- and off-target editing in an hPSC line, specifically WA09 cells (also referred to as H9s—Figure 3A). We transduced these cells with the same Cas9-d system and delivered sgRNA into these cells via nucleofection with a published protocol designed for hPSCs.[73]

The *VEGFA* site 1 sgRNA (Figure 3B), has been previously shown to be a promiscuous sgRNA,[72,74,75] with a high propensity for generating off-target indels compared to the intended edit. Hence, this sgRNA was utilized to evaluate the off-target effects in WA09 hPSCs with the Cas9-d system (referred to as Cas9-d cells). To target the *VEGFA* locus, Cas9-d cells were nucleofected with this sgRNA. Cas9-d cells were either not nucleofected, nucleofected with POM, or nucleofected without POM. Following nucleofection, cells were undisturbed for a 15-min incubation period after which designated cells were introduced to POM. All cells were left undisturbed for 48 h, after which genomic DNA was extracted. The genomic DNA was amplified via PCR and sequenced via NGS, with subsequent analysis at the on-target site and the top three off-target sites using CRISPResso2. A 3-fold decrease in editing activity at the *VEGFA* locus was observed in POM cells compared to their non-POM counterparts (Figure 3B); the on-target indel frequency was 9.31 ± 1.23% in Cas9-d cells without POM and 2.80 ± 0.876% in Cas9-d cells with POM (Figure 3B). At the three distinct off-target sites, we observed a similar trend in editing activity (Figure 3C). Adding POM to cells led to a decrease in indel frequency, while higher editing was noted in cells when Cas9-d was not subjected to degradation through POM addition.

Cas9-d efficiency in clinically relevant cells

Given that hepatocytes represent the predominant cell type in the liver for many gene therapy programs,[2,76–78] we evaluated the activity of Cas9-d within two hepatocyte cell lines (Huh7 and HepG2—Figure 4A) for the editing of the *PCSK9* gene. Base editing and knockout of *PCSK9* has been shown to lower cholesterol levels.[79–82] The cut site for the *PCSK9* gene is shown in Figure 4A. Adherent hepatocytes were transduced with media containing POM and virus; media was changed the day after with POM, and cells were sequenced after 7 days (Figure 4A). At the *PCSK9* on-target site the indel frequency revealed a dose-dependent effect (Figure 4B). Without POM treatment, there was 1.02 ± 0.500% or 1.59 ± 0.121% editing in Cas9-d cells, compared to 0.410 ± 0.0306% or 0.547 ± 0.108% editing in 100 μM POM-induced Cas9-d cells, in HepG2 cells and Huh7 cells, respectively (Figure 4B). Up to a 3-fold decrease in editing was observed in POM-induced Cas9-d hepatocytes (Figure 4B). These editing observations are consistent with those seen in HEK293T cells and WA09 hPSCs. Furthermore, cells from which 100 μM POM was withdrawn for 5 days restored editing to levels without POM (0.960 ± 0.103% and 1.07 ± 0.207% for HepG2 and Huh7 cells, respectively [Figure S4]). This 2-fold increase in editing for the POM withdrawal samples demonstrates the reversible editing activity of Cas9-d.

Finally, we transduced human neurons derived from iPSCs (iPSC-neurons) with Cas9-d and a sgRNA targeting the amyloid precursor protein (*APP*) locus[83] to evaluate Cas9-d effectiveness in a major cell type found within the central nervous system (CNS) (Figure 5). The derivation procedure produces an enriched culture of GABAergic neurons, which respond to the amino acid, gamma-aminobutyric acid (GABA), the primary inhibitory neurotransmitter in the CNS. Moreover, the *APP* gene is well known to have a role in Alzheimer's disease, making it a clinically relevant gene to target for CNS applications. After transducing iPSC-neurons with Cas9-d, we added POM to the culture media (Figure 5A). After 7 days of editing with or without POM, we observed dose-dependent on-target editing at the *APP* locus. On-target editing reached 24.3 ± 1.45% without POM but dropped 9.3-fold to 2.61 ± 0.152% with 10 μM POM (Figure 5B).

Figure 2. Assessing on-target genome editing efficiency with Cas9-d in human embryonic kidney cells

(A) On-target genome editing at the *AAVS1* locus within HEK293T cells after lipofection and treatment with POM. HEK cells contain the wild-type Cas9 without the degron (WT Cas9) or the Cas9-d system. Indel profiles after lipofection for Cas9-d and POM-induced Cas9-d at the *AAVS1* locus in HEK cells. The dotted line represents the cut site. POM-induced Cas9-d indel profile shows shorter and less deletions than no POM counterparts. The *AAVS1* locus on-target data was used to determine the modification frequency of each base pair around the cut site relative to the WT sequence. Each amplicon was divided by the number of reads not aligned with the wild-type sequences at each base pair. There is up to 4-fold decrease in indel frequency when there is POM-induced degradation of Cas9-d ($n = 3$). (B) The degron does not hinder on-target Cas9 activity and POM treatment in Cas9-d cells reduces editing by 5-fold. Data represented in bar graphs are represented as mean ± SEM, $n = 3$ technical replicates per condition, p values generated by one-way ANOVA; ns = $p \geq 0.05$ (not shown), *$p < 0.05$, **$p < 0.01$, ***$p < 0.001$, and ****$p < 0.0001$.

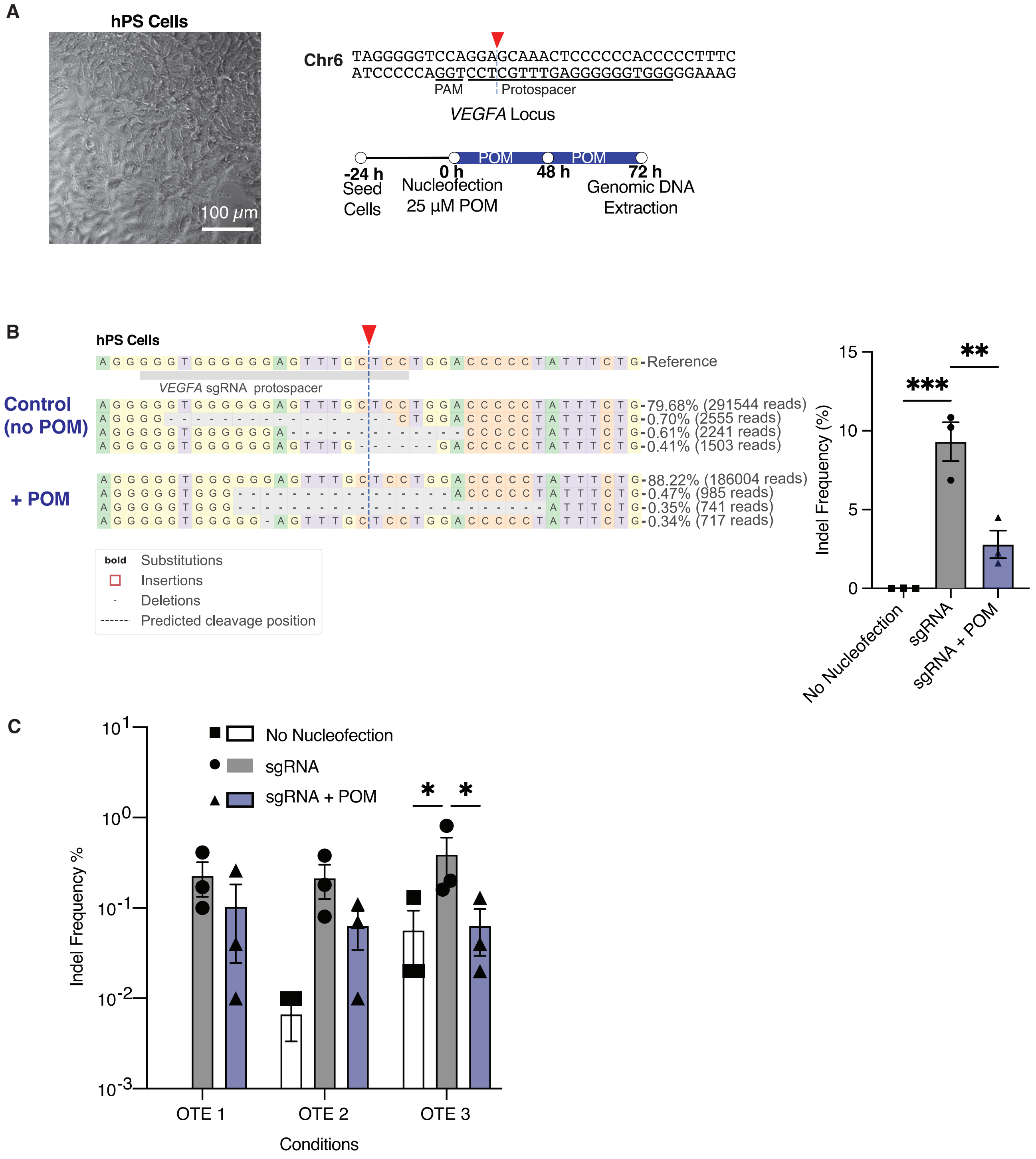

Figure 3. Evaluation of on-target efficiency and off-target indel frequency of Cas9-d in human pluripotent stem cells

(A) Representative image of edited hPSCs (WA09). Scale bar, 100 μm. Schematic of the *VEGFA* guide (underlined) and cut site (red arrow). (B) On-target genome editing at the *VEGFA* locus within WA09 cells after nucleofection shows there is a 3-fold decrease in editing at the on-target in POM treated cells when compared to the non-POM treated cells. Data represented in bar graphs are represented as mean ± SEM, $n = 3$ technical replicates per condition, p values generated by one-way ANOVA; ns = $p \geq 0.05$ (not shown), *$p < 0.05$, **$p < 0.01$, and ***$p < 0.001$. (C) Off-target editing in WA09 while targeting the VEGFA locus. Off-target editing at three different sites (OTE1, OTE2, and OTE3) show similar decrease in editing with POM as on-target editing. Data represented in bar graphs are represented as mean ± SEM, $n = 3$ technical replicates per condition, p values generated by one-way ANOVA; ns = $p \geq 0.05$ (not shown) and *$p < 0.05$.

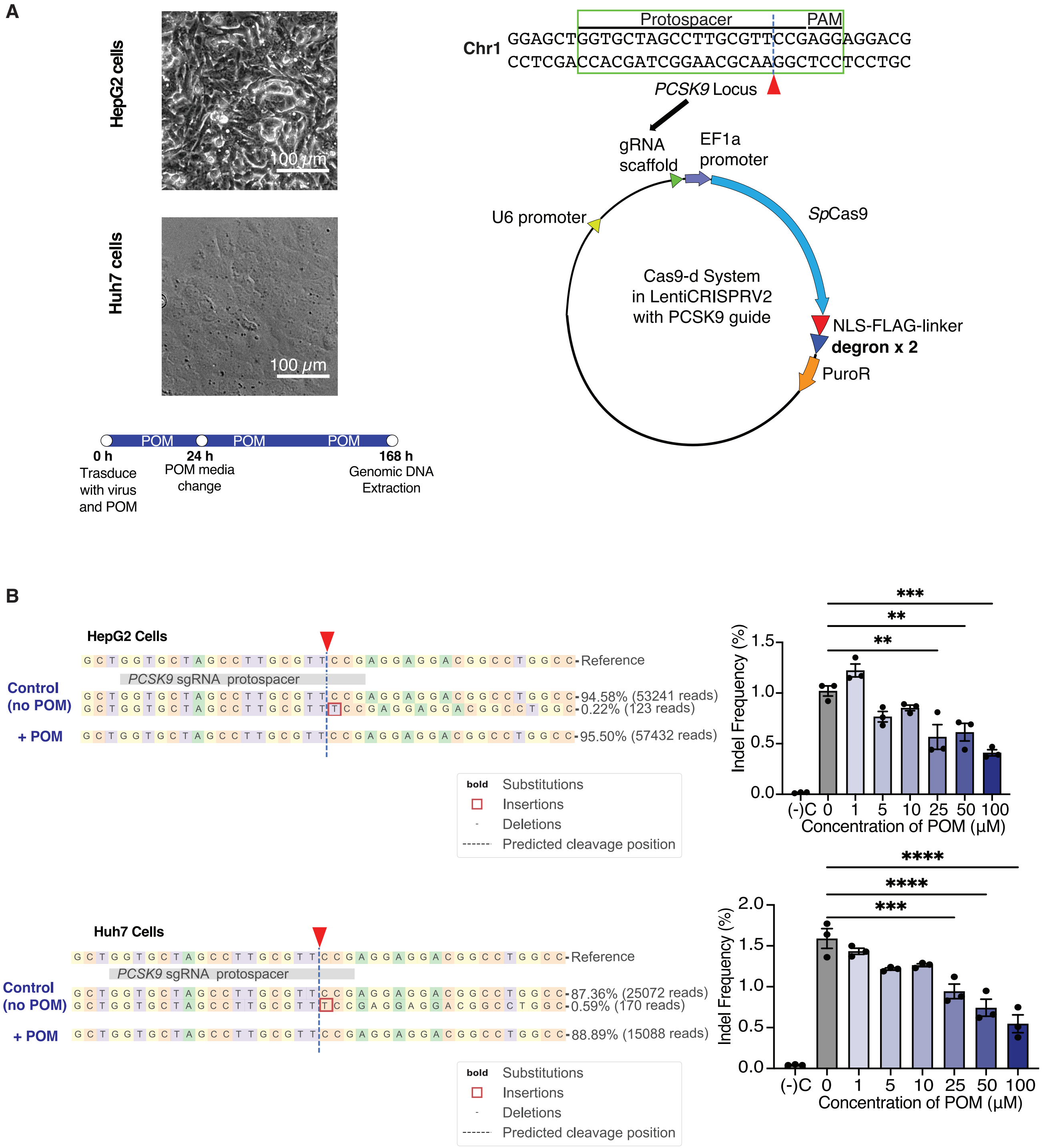

Figure 4. On-target editing at different POM doses in human hepatocyte lines

(A) Representative image of hepatocyte transduction with Cas9-d targeting the *PCSK9* locus. Schematic of the *PCSK9* locus and where the guide RNA is in the plasmid. The cut site is represented by a red triangle. Cells were transduced with virus and media, media was changed a day later, and cells continued to be cultured for a total of 7 days

(legend continued on next page)

Previous indel profiles at the *APP* on-target site in undifferentiated hPSCs[83] reveal that one of the most frequent indels results from microhomology-mediated end-joining (MMEJ), typically ranging from 5 to 20 bp. The highest frequency read was a 5-bp deletion around the predicted double-stranded break, followed by lower frequencies of 2-bp deletion, 4 bp-deletion, 1-bp deletion, and a 6-bp deletions. Unlike these previous results in hPSCs, our results showed the top read was repaired via non-homologous end-joining (NHEJ) in iPSC-neurons (6.87%) without POM and via MMEJ (0.85%) with POM (Figure 5B). NHEJ (2-bp and 1-bp deletions) was the primary repair pathway, producing 15.4% of the top reads without POM, decreasing to below 1% with POM. In contrast, the frequency of the MMEJ 5-bp deletion was 4% without POM, decreasing to below 1% with POM. We conducted several experiments under different transduction and culture conditions for iPSC-neurons, yielding reproducible results with a decrease in genome editing at the on-target site upon POM addition.

To evaluate the effects of POM on hiPSC-neuronal function, we subjected the hiPSC-neurons with Cas9-d to a high-throughput electrophysiology assay. We generated the Na^+ and K^+ current profiles from these neurons using the voltages and pulse codes found in Figures 5C and S6A, respectively. K^+ current elicited using a voltage-clamp protocol did not show any significant difference ($p = 0.16$) in the current amplitude at +60 mV between transduced hiPSC-neurons without POM treatment (182.92 ± 54.80 pA) and untreated hiPSC-neurons (299.62 ± 58.17 pA). Compared to untreated neurons, a difference ($p = 0.03$) was observed in K^+ current amplitude at +60 mV for transduced hiPSC-neurons with POM treatment (119.72 ± 43.17 pA). The normalized current (I/I_{max}), evoked by the same voltage steps, in Figure S6B, shows the reversal potential of transduced hiPSC-neurons with POM treatment ($n = 12$) and transduced with no POM treatment ($n = 13$) is shifted toward depolarizing potential (−20 mV) compared to untreated hiPSC-neurons (−60 mV, $n = 17$). Normalized conductance (G)-voltage (V) curve (fitted using a sigmoidal function, $R^2 = 0.99$), showed a significant difference ($p < 0.0001$) in activation potential of transduced and treated neurons (POM and no POM) compared to untreated hiPSC-neurons (13.17509 ± 1.94896) (Figure S6C). Moreover, the activation potential of no POM (−12.35971 ± 2.05638) vs. POM-treated (−16.4009 ± 2.7058) transduced hiPSC-neurons was also significantly different ($p = 0.003$). The Na^+ channel current profile was not significantly different among transduced hiPSC-neurons treated with and without POM compared to untreated hiPSC-neurons (Figure S6D).

DISCUSSION

By controlling the levels of *Sp*Cas9 nuclease over the timescale of 6–72 h with POM, we could tune on-target editing efficiency *in vitro*. The degron tag used in Cas9-d did not compromise the gene editing activity of the Cas9 nuclease, and repeated administration of POM did not substantially further alter the degradation kinetics of the Cas9 protein. One dose of POM in the culture media decreased Cas9 protein levels for an extended duration, up to 96 h. Plus, genome editing efficiency and precision were restored when POM was withdrawn. Such reversibility provides a convenient toggle switch for genome editing *in vitro*, allowing for the gradual editing of cells by turning editing on and off. In comparison to the 10–20 h half-life of native Cas9 ribonucleoprotein (RNP),[84] the Cas9-d system achieved a reduced half-life of 7.31 h in HEK293T cells. The reduced half-lives of Cas9-d nucleases led to a 3- to 5-fold decrease in Cas9-d activity at endogenous on-target sites across several cell lines, including cancer, embryonic stem, and mature cells.

Complete elimination of Cas9-d protein via POM would be an ideal scenario to abolish nuclease activity on demand; however, this is a challenge in our culture systems with human cells. In HEK293T cells, at least 20% residual Cas9-d protein persists after 96 h (Figure 1F), preventing Cas9-d levels from reaching 0%. This challenge arises from three potential mechanisms. First, the Cas9-d system in this study relies on the balance between transcription and degradation. Because Cas9-d continues to be transcribed and translated from a constitutive promoter, degradation must occur faster than translation. A potential solution to this problem is to lower the expression level of Cas9-d or to modulate the dynamics of the Cas9-d expression in a tunable manner to match the degradation rate of the proteasomal system. Second, the time required to shuttle Cas9-d proteins to the proteasome may be substantial enough for Cas9 to continue nuclease activity. Previous studies indicate that it can take anywhere from several minutes to several days for certain proteins to be degraded by the proteasome,[85] but none provide a definitive answer on the precise duration between ubiquitination and the targeting of the protein to the proteasome. Third, off-target neo-substrates of POM can compete for the CRBN E3 ligase substrate receptor pool.[86] Immunomodulatory drugs (IMiDs) such as POM have been found to have numerous off-target neo-substrates, which are proteins degraded in the presence of IMiDs by the CRL4-CRBN complex.[87,88] This target competition between other neo-substrates and Cas9-d could lead to a faster depletion of CRBN and POM in human cells.

Neo-substrates of IMiDs include IKZF1, IKZF3, CSNK1A1, ZFP91, GSPT1, RNF166, ZNF692, GSPT2, ARID2, and SAL4.[86,89–94] Using the Human Protein Atlas,[95,96] we compiled the protein levels and RNA expression levels of these neo-substrates in multiple organs that could be edited *in vivo* (brain, kidney, liver, and eye) as well as cell lines derived from these tissues (inhibitory neurons, HEK293T, HepG2, Huh7, and hTERT-RPE1; see Table S3). We

before genomic extraction. (B) On-target editing at the *PCSK9* locus in HepG2 cells and Huh7 cells expressing Cas9-d. POM (1 μM) is insufficient to decrease on-target indels, but at higher doses, there is a dose-dependency in producing on-target indels. Moreover, there is a 2- to 3-fold decrease in editing when comparing 0 μM and 100 μM POM samples. Data represented in all bar graphs are represented as mean ± SEM, $n = 3$ technical replicates per condition, p values generated by two-way ANOVA; ns = $p \geq 0.05$ (not shown), *$p < 0.05$, **$p < 0.01$, ***$p < 0.001$, and ****$p < 0.0001$.

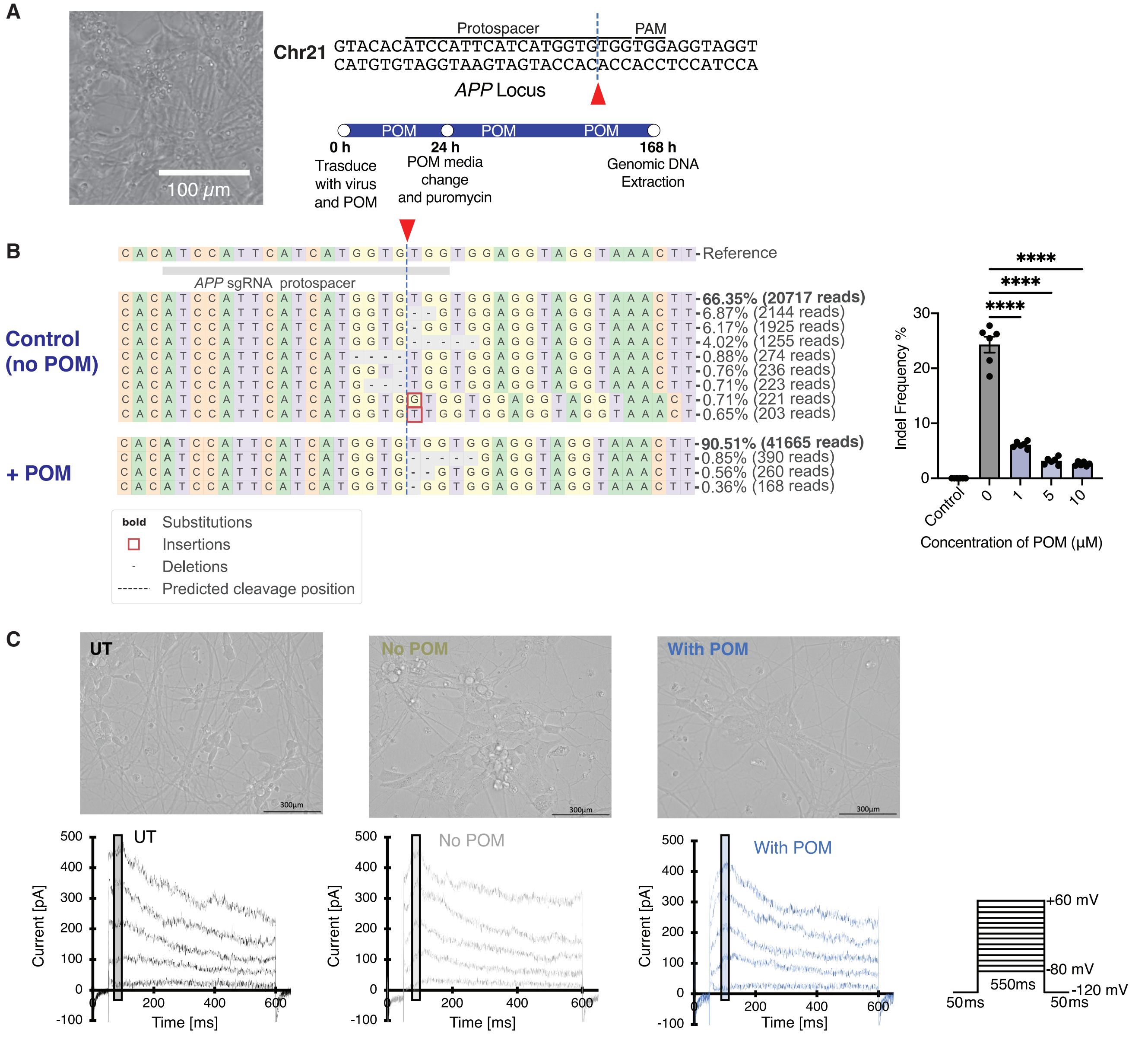

Figure 5. High-efficiency genome editing in human hiPSC-derived cortical GABAergic neurons

(A) Representative image of neurons. Neurons were transduced with Cas9-d virus while in suspension with POM media on day 0. On day 1, there was a full media change, followed by half a media change on day 4, and genomic DNA extraction of day 7. (B) Indel profiles after transduction for Cas9-d and POM-induced Cas9-d at the *APP* locus in neurons. The dotted line and red arrow represents the cut site. POM-induced Cas9-d indel profile show less deletions than no POM counterparts. There is a 4.8-fold decrease in editing when cells are POM-induced with 1 μM POM. Data represented in bar graphs are represented as mean ± SEM, $n = 5$ technical replicates per condition, p values generated by two-way ANOVA; ns = $p \geq 0.05$ (not shown), *$p < 0.05$, **$p < 0.01$, ***$p < 0.001$, and ****$p < 0.0001$. (C) Representative image of untransduced neurons, neuron transduction with Cas9-d with and without POM targeting the *APP* locus. Step evoked K current profile in transduced and POM treated neurons with respect to untreated neurons. The current is elicited by voltage protocol shown on the bottom right. The stimulation protocol consisted of a holding potential (Vh) of −120 mV with 10 mV voltage steps from −80 mV to +60 mV, then back to Vh.

did not find any protein-related information on any neo-substrates or cereblon (one of the components involved in degradation machinery) for the retina, which is the target of many genome-editing strategies.[97] In contrast, ZFP91 proteins are highly expressed in the kidney, liver, and neuronal cells, while RNA expression of CSNK1A1 and GSTP1 is highly expressed in inhibitory neurons, HEK293T, HepG2, and Huh7 cells. Characterizing the states and activity of CSNK1A1 and GSTP1 within targeted brain and liver cells could

be essential to monitor safety studies in future preclinical and translational work with the Cas9-d system as POM interacts with these neo-substrates. In tissues with many neo-substrate proteins, the new IMiD analog, PT-179, controls a superdegron called SD40[98] and is predicted to have fewer neo-substrates and overall gene expression perturbations than POM.[98] Incorporating SD40 into editors and triggering degradation via PT-179 could effectively eliminate editing activity. However, PT-179 may have a different biodistribution and safety profile than POM, which must be characterized for *in vivo* work, especially for cellular targets within the blood-brain barrier.

Our studies identify optimal dosing ranges for POM for use with the Cas9-d system. A previous study shows that 10 μM POM *in vitro* has no significant effect on cellular viability.[48] Our studies began to show toxicity in some cells at 50 μM POM. Hence, we recommend a range of 1–25 μM POM for our *in vitro* studies, noting that 1 μM POM had cell-type effects on Cas9-d activity. Clinical studies have shown that doses ranging from 2 to 4 mg have been safely administered to patients with multiple myeloma, and up to 5 mg has been used in participants with Kaposi sarcoma.[99,100] Although POM has a promising safety profile in patients, it may still have subtle, tissue- and cell-line-specific effects that need to be addressed in future work by developing other POM analogs or by further protein engineering the binding region in the degron.

Because POM is known to cross the blood-brain barrier, the Cas9-d system may be a valuable tool for CNS-targeted therapies.[48,101] The genomic outcomes at *APP* from Cas9-d aligned with previous findings on *Sp*Cas9. Sun et al. demonstrated that in hPSCs, transient co-expression CRISPR-Cas9 and the *APP*-sgRNA led to genomic deep sequencing results showing the most common mutation as a 5-bp deletion with 81.8% frequency, followed by 2-bp deletion at 0.3%, and 4-bp, 1-bp, and 6-bp deletion each at 0.2% frequency.[83] Notably, MMEJ editing of *APP* produces a C-terminal truncation shown to be protective against Alzheimer's disease,[102] and we observe this particular 5-bp MMEJ result with Cas9-d in our iPSC-neurons. Two main pathways of repair take place due to CRISPR-Cas9 editing when there is no donor template present: NHEJ or MMEJ. The kinetics of DNA repair have been profiled after RNP-mediated double-stranded DNA cleavage in hPSCs[103]: 50% of NHEJ-mediated insertions occurred by 5.3 h after delivery, while 50% of MMEJ-mediated deletions occurred later, by 16.1 h. In our experiments, 20% of the outcomes in the no POM condition are MMEJ-based even though NHEJ is the predominant repair pathway in non-dividing cells.[104] Although we cultured cells in BrainFast SK media, specifically designed to eliminate dividing non-neuronal cells, we cannot rule out the possibility of residual non-neuronal cells in our culture, which may be more proliferative and undergo MMEJ for DNA repair preferentially. Developing a separate lentiviral construct driven by a neuron-specific promoter with the Cas9-d system could confirm that the editing outcomes arise exclusively in iPSC-neurons. Our editing data in iPSC-neurons, shown in Figure 5B, demonstrate dose-responsive editing following POM treatment, consistent with Cas9 degradation being the primary mechanism of action. Additionally, we closely monitored cell viability to eliminate general cytotoxic effects of POM as a confounding factor and did not observe increasing toxicity in our cultures with higher POM concentrations.

The half-life of the RNP is known to be ∼20 h[84] In comparison, our POM-induced Cas9-d technology demonstrates a superior degradation half-life of 7.31 h, even as Cas9-d is continuously synthesized. This is an improvement over the SMASh-Cas9, which has a half-life of 10.6 h and is transiently expressed from a plasmid.[47] The Cas9-d is currently a system that turns "off" when POM is added, so additional control of Cas9-d could be added to provide more control over Cas9 levels. The X^{on} cassette system may provide additional control over Cas9-d and act as an on-switch. Monteys et al. developed the X^{on} system, which helps control alternative splicing via FDA-approved blood-brain barrier crossing small molecules, such as branaplam (LMI070).[105] They report that a miniX^{on} system with SaCas9 and a guide RNA that targeted the loxP-stop cassette in Ai14 mice had a nonsignificant reduction in activity compared to the X^{on} system.[105] Just like our Cas9-d system, the miniX^{on} system does not require the co-expression of any regulatory or exogenous proteins and is considerably small at ∼186 amino acids long. Another promising approach is to combine our degron-based Cas9-d system with the 4-OHT-regulated split Cas9,[26,32] as the 4-OHT-based system offers tighter control and lower background activity. A hybrid split Cas9-d design could offer both tight temporal regulation and reduced off-target activity. Additionally, engineering a degron-sensitive 4-OHT-Cas9 fusion would enable orthogonal small-molecule control over activation and deactivation kinetics, such as the X^{on} Cas9-d system. Moreover, incorporating the degron into synthetic mRNA encoding Cas9 or RNPs could also enable finer control of Cas9 activity via dosing strategies. Complete degradation of dead Cas9 was achieved in RNP formats with a similar degron at POM concentrations as low as 100 nM within an hour of treatment.[106] Future research in these areas could help achieve a balance between precision and reversibility in therapeutic genome editing applications.

Aside from the expression and degron systems to further reduce half-life, different formats and delivery methods could be used in future therapeutic applications upon further development. For viral delivery, cell-specific promoters (e.g., synapsin for neuronal specificity)[107] and other vectors (e.g., AAV vectors and non-integrating lentiviral vectors)[35,108] are likely necessary for clinical development in many therapeutic applications. Furthermore, the transient delivery of a Cas9 RNP-degron complex, rather than sustained expression, could also establish tighter temporal control for therapeutic applications. Nonviral delivery vectors could layer on spatiotemporal control of the release of these degron-containing mRNA or RNP payloads through the use of near-infrared light-triggered photothermal release of Cas9 from various types of nanoparticles,[109–112] ultrasound-controlled CRISPR-Cas9 nanosystem,[113] pH-responsive polymer nanoparticles,[114] and glutathione-responsive nanoparticles.[115,116] Other strategies can control the release of sgRNA via near-infrared[117]

and UV light.[118] There are several promising methods to layer more control of CRISPR-Cas9 with the degron in a combinatorial fashion.

CONCLUSIONS

Relative to prior methods[47,54] to control Cas9 nuclease activity with small molecules, we show that targeting Cas9 protein for degradation removes active Cas9 enzymes in human cells within hours after adding an FDA-approved drug, POM. Since lentiviral vectors have been used in several clinical trials,[61–64] the vectors described here could be further developed for cell and gene therapy, potentially with alternative vectors such as AAV that can package the Cas9-d system with small Cas9 homologs, such as *Sa*Cas9[119] and *Nme*Cas9.[120,121] Further development of the Cas9-d system could refine *in vivo* gene editing strategies and *ex vivo* cell therapies precisely edited by Cas9-d. This experimental *in vitro* approach improves the genome editing toolkit, paving the way for new biomanufacturing and synthetic biology directions.[122–124] Notably, precise temporal regulation of Cas9 gene editing activity *in vitro* could be particularly valuable for investigating genome editing kinetics and safety in various human-based platforms that are gaining traction for regulatory decision making.[125] Degrons similar to this one have already helped control chimeric antigen receptors on T cells,[126,127] in screening applications,[128] and degron-based control could enable multiplexed editing (targeting multiple genes) with reduced risk of off-target effects.[129–131] Moreover, this degron system can be adapted for use with other genome editors, such as zinc-finger nucleases,[132] transcription activator-like effector nucleases,[133] base editors,[106] epigenomic editors,[134,135] and other CRISPR-Cas systems in development.[136–138] Ongoing development to control the lifetime of genome editors within cells will enhance our understanding of the mechanisms involved in genome editing and refine the activity to be more accurate, safe, and versatile.

MATERIALS AND METHODS

Cell culture

All cells (HEK293T, WA09, Huh7, HepG2, and hiPSC-derived GABAergic neurons) were maintained at 37°C and 5% CO_2. Depending on the specific experiments, HEK293T, WA09, Huh7, and HepG2 were seeded at a density of 200,000 cells per well on 6-well plates, 100,000 cells per well on 12-well plates, 50,000 cells per well on a 24-well plate, or 20,000 cells on a 96-well plate (Corning, Corning, NY). HEK293T cells were cultured with media composition of 90% DMEM, high glucose (Gibco, no. 11965118, Waltham, MA), 9% fetal bovine serum (FBS) (Gibco, no. 16000044, Waltham, MA), and 1% penstrep (Gibco, no. 15140122, Waltham, MA). WA09 and AICS-61 cells were seeded on Culturex (R&D Systems, no. 3434, Minneapolis, MN) coated plates with mTeSR1 (STEMCELL Technologies, no. 85850, Vancouver, Canada) media. Huh7 and HepG2 cells were cultured with media containing 90% DMEM F-12 (Gibco, no. 11330-032, Waltham, MA), 9% FBS, and 1% penstrep. Neurons were seeded at 50,000 cells per well on a 96-well plate or 500,000 cells per well on a 6-well plate and handled with media according to the manufacturer's instruction.[139]

Guide RNA cloning

Integrated DNA Technologies (IDT) synthesized the 25-bp guide sequence with the right overhangs and 5′ phosphorylation. These oligonucleotides contain protospacer sequences complementary to the target gene and appropriate overhangs for cloning. Next, 5 μg of either lentiCRISPR v2 (Addgene, no. 52961, Watertown, MA) or lentiCRISPRv2-mCherry (Addgene, no. 99154) was digested with 3 μL of *BsmBI*-V2 (NEB, no. R0739, Ipswich, MA), 6 μL of 10× enzyme buffer provided with the enzyme, freshly prepared 100 mM dithiothreitol (Thermo Fisher Scientific, no. R0861, Waltham, MA), and ribonuclease (RNase)-free water up to 60 μL. BsmBI-v2 was used according to the manufacturer's instructions. Dephosphorylation of the digested vector was done with Quick CIP (NEB, no. M0525) according to the manufacturer's instructions. Subsequently, the digested vector was purified using the QIAquick PCR Purification Kit (QIAGEN, no. 28104, Germantown, MD) and eluted with a warm elution buffer. The oligonucleotides were resuspended to 100 μM and mixed in equimolar concentrations with annealing buffer (10 mM Tris [pH 7.5–8.0], 50 mM NaCl, and 1 mM EDTA) (made in-house), constituting 10% of the final concentration. The annealing reaction was conducted in the thermocycler using the following parameters: 95°C for 2 min, ramp down 0.08°C/s to 25°C for 45 min, hold at 4°C. Subsequently, the vector (50 ng) and guide RNA oligonucleotides (0.29 ng) were mixed and ligated together using Quick Ligase (NEB, no. M2200) as per the manufacturer's directions. Following ligation, manufacturer instructions transformed the mixture into One Shot Stbl3 Chemically Competent *E. coli* (Invitrogen, no. C737303, Waltham, MA). Colonies were grown and sequenced to confirm the correct insert.

Lentivirus production

A day before transfection, human embryonic kidney (HEK) 293T cells were seeded in 4 T–75 flasks (Thermo Fisher Scientific, no. 156499) at a density of 4.5 × 10^6 cells per flask in HEK media. After 1–2 days, when cells reached 70%–90% confluency, transfection was carried out using Lipofectamine 2000 (Invitrogen, no. 11668019). Sixty microliters of Lipofectamine 2000 were diluted in 1 mL of Opti-MEM (Gibco, no. 31985062, Waltham, MA) and incubated for 5 min. Next, 10 mg of the LentiCRISPR v2 plasmid with a guide, 2 μg of pMD2.G (Addgene, no. 12259), and 5 μg of psPAX2 (Addgene, no. 12260) were mixed with 1 mL of DMEM. After incubating the lipofectamine mixture with DNA for 30 min at room temperature, it was added to 8 mL of media (DMEM+FBS, no P/S). The sample was then incubated at 37°C and 5% CO_2 overnight, and the media was changed to 10 mL of DMEM+FBS+P/S the next day. The supernatant was collected on day 5 and day 6, spun down at 300 × g for 5 min, and transferred to a new tube without disturbing the cell debris pellet. On day 6, the Lenti-X Concentrator (Takara Bio, no. 631231, San Jose, CA) was added equal to 1/3 of the supernatant for both day 5 and day 6 supernatants. On day 7, samples were spun down at 4°C at 1500 × g for 45 min. The supernatant was discarded, and the pellet was resuspended in PBS to make 100 μL stocks and frozen in cryovials.

Viral titer

The viral titer was quantified using Abcam's Lentivirus qPCR Quantification Kit (Abcam, ab289841, Waltham, MA), in addition to antibiotic selection methods. Sample preparation followed manufacturer's instructions. Using the manufacturer's recommendations, the multiplicity of infection (MOI) for virus was determined using volumes ranging from 1 to 10^{-6} μL virus. The viral titer for *PCSK9* mCherry lentiCRISPR was 5.75×10^7 IU/μL, for LentiCRISPR Cas9-d with a PCSK9 sgRNA was 3.51×10^7 IU/μL and for LentiCRISPR Cas9-d with *APP* sgRNA was 8.71×10^7 IU/μL. To assess the titer through antibiotic selection, cells were plated to nearly confluence and amount which had priorly been collected through kill curve was used. Dead/alive cells were estimated within the next two days and determined that 10^{-3} μL virus had an MOI of 0.35 versus cells in μL to 10^{-2} μL virus had no death. Therefore, the lower MOI of 0.35 was chosen to be used as it also correlated with the actual viral titer.

Hepatocyte transduction

Both Huh7 and HepG2 hepatocyte lines underwent transduction using the same method as follows. Cells were plated in triplicates into a 24-well plate at a density of 50,000 cells a day before virus addition. The next day, media was changed to 200 μL of DMEM-F12+FBS supplemented with polybrene (8 μg/mL) for 3 h with LentiCRISPR Cas9-d with a sgRNA targeting the *PCKS9* locus and maintained at 37°C and 5% CO_2. After 3 h, 300 μL of media was added.

hiPSC-derived cortical GABAergic neuron transduction

GABAergic neurons (BrainXell, BX-0400-30, Madison, WI) were maintained at 37°C and 5% CO_2, seeded at 50 K/well and handled according to the manufacturer's instruction.[139] Cells were transduced while seeding (as per manufacturer's instructions) at MOIs of 0.5, 1, or 2 with LentiCRISPR Cas9-d with a sgRNA targeting the *APP* locus. POM amounts of 0, 10, or 25 μM were added. Media was completed changed the next day with Basal Media plus 1× BrainFast GABA (BrainXell, no. BX-2400) and 1× BrainFast SK (BrainXell, no. BX-2020).

Cell viability assay

Cell-based assays are usually used to determine if specific reagents or molecules may be affecting cell proliferation or viability.[140] The 0.4% trypan blue dye (Invitrogen, no. T10282) exclusion test was used as a quick method to determine the number of viable cells after the different titrations of POM (Selleckchem, no. S1567, Houston, Texas). Live cells with intact cell membranes did not take up the dye, while nonviable cells took up the dye and displayed blue cytoplasm. 24 h before addition of either 0, 1, 10, 25, 50, or 100 μM of POM, cells were seeded at 200,000 cells per well on a 6-well plate. Different quantities of POM were added to triplicates of wells and 24 h later, cells were trypsinized and counted. Additionally, cells were passaged and then 24 h later counted again. The following equation was used to determine the amount of POM to use for the cells when conducting future experiments:

viable cells % = # of viable cells per ml of aliquot / # of total cells per ml of aliquot x 100

Delivery of sgRNA via lipofection

HEK293T were singularized with trypsin and counted using the Countess II FL Automated Cell Counter (Thermo Fisher Scientific) with 0.4% Trypan Blue viability stain (Invitrogen, no. T10282). HEK293T cells were seeded at a density of 20,000 cells per well on a 96-well plate, two days prior to lipofection. One hour prior to lipofection, HEK293T cells were treated with 25 μM POM in fresh media. Lipofection was performed with 0.5 μL Lipofectamine 2000 (1000 ng Cas9/well and 1:1 crRNA:tracrRNA). Cells were undisturbed for 48 h and then genomic DNA was extracted for downstream analysis. The sgRNA sequences are in Table S1.

Delivery of sgRNA via nucleofection

For hPSC electroporation experiments, the Amaxa 4D-Nucleofector by Lonza was used as per manufacturer's instructions. H9s were singularized using Accutase (STEMCELL Technologies, no. 07920, Vancouver, Canada) and counted using the Countess II FL Automated Cell Counter with 0.4% Trypan Blue viability stain. Subsequently, 200,000 cells per electroporation underwent centrifugation at 300 × g for 5 min. After removing the media carefully, without disturbing the pellet, cells were resuspended in a solution mix consisting of 21 μL of P3 solution (Lonza, no. V4XP-3032, Walkersville, MD) and 2 μL of 100 μM sgRNA per nucleofection. iPSCs were nucleofected using the program CB-150. Post-nucleofection, cells were incubated for 15-min and then plated in a 96-well plate containing mTeSR1 media supplemented with 10 μM ROCK Inhibitor (STEMCELL Technologies, no. 72302). Additionally, 25 μM POM was introduced at varied intervals as indicated in the figures. Media replacement with mTeSR1 occurred 48 h post-transfection, following which the cells remained undisturbed for an additional 24–48 h. Genomic DNA extraction was then extracted for downstream analysis.

Deep sequencing

Genomic DNA was extracted from various cells on different days using QuickExtract/well (Epicentre). Quick extract and genomic DNA was incubated at 65°C for 15 min, 68°C for 15 min, and 98°C for 10 min. We targeted PCR amplification around the different loci of interest using Q5 Hot Start Polymerase (NEB) according to manufacturer's instructions and primers in Table S2. During the second round of PCR, we added unique dual indices (IDT), followed by an AMPure XP magnetic bead clean-up (Beckman Coulter). The samples were pooled together and sequenced on the Illumina MiniSeq at a run length of 1 × 150 bp according to the manufacturer's instructions. We performed analysis using CRISPResso2 and GraphPad Prism 9.

Western blot analysis

To evaluate the expression of the Cas9 protein, western blotting, a method to semi-quantify the presence, relative abundance, relative

mass, and presence of post-translational modifications,[141] was used. Cells expressing Cas9-degron with or without POM were isolated and lysed using Pierce radioimmunoprecipitation assay buffer (Thermo Fisher Scientific, no. 89900) supplemented with 1× protease inhibitor and 1× phosphatase inhibitor (Thermo Fisher Scientific, nos. 78429 and 78420, respectively). Protein concentrations were determined through Lowry protein assay (Bio-Rad DC Protein Assay, Hercules, CA). Protein lysate (20 μg) was loaded onto 4%–20% Mini-PROTEAN TGX Precast Gel (Bio-Rad, no. 4561094, Hercules, CA) and transferred to polyvinylidene fluoride membranes (Thermo Fisher Scientific, no. 88518), and blotted with a rabbit anti-Cas9 antibody (1:1000) (Takara Bio, no. 632600) or mouse anti-tubulin antibody (Cell Signaling Technologies, no. 3873, Danvers, MA) overnight at 4°C. Washes were conducted the next day, and membranes were incubated in horseradish peroxidase-conjugated goat anti-rabbit (Jackson ImmunoResearch Inc., no. 111-035-003, West Grove, PA) or goat anti-mouse (Jackson ImmunoResearch Inc., no. 115-035-003) immunoglobulin IgG (respectively) at room temperature, followed by addition of enhanced chemiluminescence substrate (made in-house). Membranes were visualized using a chemiluminescence imager.

Electrophysiology assay

The automated patch clamp (Q Patch II, Sophion) was used to measure whole-cell sodium (Na) and potassium (K) channels from iPSC-neurons that were either untransduced (n = 17), transduced without POM (n = 13), or transduced with POM (n = 12). The cells were transduced 2 weeks prior to measurements and after 2× PBS washes, detached gently using Accutase for 15 min. Cells were neutralized with 3 mL of serum-free medium containing 25 mM HEPES, and gently pipetted to release them from the PDL-coated plates. Cells were centrifuged at 100 × g for 2 min and resuspended in 1 mL of serum-free medium. To enhance single-cell separation, the cells were kept on a shaker for 20 min. A pressure protocol was used to achieve cell positioning, giga-cell and whole-cell as described earlier.[142] To isolate K^+ channel currents, the current was recorded in response to voltage-clamp steps from the holding potential (−120 mV) to voltages between −80 mV and +60 mV (Δ = 10 mV). To generate Na^+ currents, voltages ranged from −90 mV to 0 mV for 10 ms. The intracellular solution contained the following reagents (mM): 120 KF, 20 KCl, 10 HEPES, 10 EGTA, pH adjusted to 7.2 with KOH, and osmolarity of 304 mOsm. The extracellular solution contained the following reagents (mM): 2 $CaCl_2$, 1 $MgCl_2$, 10 HEPES, 4 KCl, 145 NaCl, 10 glucose, with pH adjusted to 7.4 with NaOH, and osmolarity 305 mOsm with sucrose. The Sophion Analyzer v.7.0.58 was used to analyze data. Data are presented as mean ± standard error.

DATA AVAILABILITY

Data are available upon request.

ACKNOWLEDGMENTS

We thank members of the Saha Lab for experimental advice. We thank Dr. Jichao Sun, Dr. Subhojit Roy, Dr. Kaivalya Molugu, Dr. Amr Abdeen, and Dr. Kirstan Gisme in helpful discussion in design of the degron and sequencing advice. We also acknowledge support from NIH T32 HG002760/HG/NHGRI (N.K.), NIH R35 GM119644-01 (K.S.), NIH/NINDS 1U19NS132296 (K.S.), and the Retina Research Foundation Kathryn and Latimer Murfee Chair (K.S.).

AUTHOR CONTRIBUTIONS

Conceptualization, N.K. and K.S.; experimental design, N.K. and K.S.; methodology, N. K. and V.P.; data analysis, N.K., V.P., and R.D.; writing – original draft, N.K.; writing – editing, N.K., K.S., V.P., R.D., M.K., and B.R.P.; supervision, K.S.; electrophysiology, M. K. performed the electrophysiology work with supervision from B.R.P.

DECLARATION OF INTERESTS

K.S. is a member of the advisory boards for Andson Biotech and Bharat Biotech.

SUPPLEMENTAL INFORMATION

Supplemental information can be found online at https://doi.org/10.1016/j.omtn.2025.102640.

REFERENCES

1. Kan, M.J., and Doudna, J.A. (2022). Treatment of genetic diseases with CRISPR genome editing. JAMA, J. Am. Med. Assoc. *328*, 980–981. https://doi.org/10.1001/jama.2022.13468.
2. Musunuru, K., Grandinette, S.A., Wang, X., Hudson, T.R., Briseno, K., Berry, A.M., Hacker, J.L., Hsu, A., Silverstein, R.A., Hille, L.T., et al. (2025). Patient-specific in vivo gene editing to treat a rare genetic disease. N. Engl. J. Med. *392*, 2235–2243. https://doi.org/10.1056/NEJMoa2504747.
3. Darle, A., Mahiet, T., Aubin, D., Doyen, M., El Kassar, L., Parfait, B., Lemaitre, G., Baldeschi, C., Allouche, J., and Holic, N. (2024). Generation of heterozygous and homozygous NF1 lines from human-induced pluripotent stem cells using CRISPR/Cas9 to investigate bone defects associated with neurofibromatosis type 1. Front. Cell Dev. Biol. *12*, 1359561. https://doi.org/10.3389/fcell.2024.1359561.
4. Huth, T., Dreher, E.C., Lemke, S., Fritzsche, S., Sugiyanto, R.N., Castven, D., Ibberson, D., Sticht, C., Eiteneuer, E., Jauch, A., et al. (2023). Chromosome 8p engineering reveals increased metastatic potential targetable by patient-specific synthetic lethality in liver cancer. Sci. Adv. *9*, eadh1442. https://doi.org/10.1126/sciadv.adh1442.
5. Samach, A., Mafessoni, F., Gross, O., Melamed-Bessudo, C., Filler-Hayut, S., Dahan-Meir, T., Amsellem, Z., Pawlowski, W.P., and Levy, A.A. (2023). CRISPR/Cas9-induced DNA breaks trigger crossover, chromosomal loss, and chromothripsis-like rearrangements. Plant Cell *35*, 3957–3972. https://doi.org/10.1093/plcell/koad209.
6. Leibowitz, M.L., Papathanasiou, S., Doerfler, P.A., Blaine, L.J., Sun, L., Yao, Y., Zhang, C.-Z., Weiss, M.J., and Pellman, D. (2021). Chromothripsis as an on-target consequence of CRISPR-Cas9 genome editing. Nat. Genet. *53*, 895–905. https://doi.org/10.1038/s41588-021-00838-7.
7. Poovaiah, C., Phillips, L., Geddes, B., Reeves, C., Sorieul, M., and Thorlby, G. (2021). Genome editing with CRISPR/Cas9 in Pinus radiata (D. Don). BMC Plant Biol. *21*, 363. https://doi.org/10.1186/s12870-021-03143-x.
8. Adikusuma, F., Piltz, S., Corbett, M.A., Turvey, M., McColl, S.R., Helbig, K.J., Beard, M.R., Hughes, J., Pomerantz, R.T., and Thomas, P.Q. (2018). Large deletions induced by Cas9 cleavage. Nature *560*, E8–E9. https://doi.org/10.1038/s41586-018-0380-z.
9. Kosicki, M., Tomberg, K., and Bradley, A. (2018). Repair of double-strand breaks induced by CRISPR-Cas9 leads to large deletions and complex rearrangements. Nat. Biotechnol. *36*, 765–771. https://doi.org/10.1038/nbt.4192.
10. Park, S.B., Uchida, T., Tilson, S., Hu, Z., Ma, C.D., Leek, M., Eichner, M., Hong, S. G., and Liang, T.J. (2022). A dual conditional CRISPR-Cas9 system to activate gene editing and reduce off-target effects in human stem cells. Mol. Ther. Nucleic Acids *28*, 656–669. https://doi.org/10.1016/j.omtn.2022.04.013.
11. Sun, N., Petiwala, S., Wang, R., Lu, C., Hu, M., Ghosh, S., Hao, Y., Miller, C.P., and Chung, N. (2019). Development of drug-inducible CRISPR-Cas9 systems for large-scale functional screening. BMC Genom. *20*, 225. https://doi.org/10.1186/s12864-019-5601-9.

12. Chen, K., Stahl, E.C., Kang, M.H., Xu, B., Allen, R., Trinidad, M., and Doudna, J.A. (2024). Engineering self-deliverable ribonucleoproteins for genome editing in the brain. Nat. Comm. *15*, 1727. https://doi.org/10.1038/s41467-024-45998-2.
13. Yang, S., Li, S., and Li, X.-J. (2018). Shortening the Half-Life of Cas9 Maintains Its Gene Editing Ability and Reduces Neuronal Toxicity. Cell Rep. *25*, 2653–2659.e3. https://doi.org/10.1016/j.celrep.2018.11.019.
14. Weisheit, I., Kroeger, J.A., Malik, R., Klimmt, J., Crusius, D., Dannert, A., Dichgans, M., and Paquet, D. (2020). Detection of Deleterious On-Target Effects after HDR-Mediated CRISPR Editing. Cell Rep. *31*, 107689. https://doi.org/10.1016/j.celrep.2020.107689.
15. Haapaniemi, E., Botla, S., Persson, J., Schmierer, B., and Taipale, J. (2018). CRISPR-Cas9 genome editing induces a p53-mediated DNA damage response. Nat. Med. *24*, 927–930. https://doi.org/10.1038/s41591-018-0049-z.
16. Sürün, D., Schneider, A., Mircetic, J., Neumann, K., Lansing, F., Paszkowski-Rogacz, M., Hänchen, V., Lee-Kirsch, M.A., and Buchholz, F. (2020). Efficient generation and correction of mutations in human iPS cells utilizing mRNAs of CRISPR base editors and prime editors. Genes *11*, 511. https://doi.org/10.3390/genes11050511.
17. Morgens, D.W., Wainberg, M., Boyle, E.A., Ursu, O., Araya, C.L., Tsui, C.K., Haney, M.S., Hess, G.T., Han, K., Jeng, E.E., et al. (2017). Genome-scale measurement of off-target activity using Cas9 toxicity in high-throughput screens. Nat. Commun. *8*, 15178. https://doi.org/10.1038/ncomms15178.
18. Zuccaro, M.V., Xu, J., Mitchell, C., Marin, D., Zimmerman, R., Rana, B., Weinstein, E., King, R.T., Palmerola, K.L., Smith, M.E., et al. (2020). Allele-specific chromosome removal after Cas9 cleavage in human embryos. Cell *183*, 1650–1664.e15. https://doi.org/10.1016/j.cell.2020.10.025.
19. Lee, B., Lee, K., Panda, S., Gonzales-Rojas, R., Chong, A., Bugay, V., Park, H.M., Brenner, R., Murthy, N., and Lee, H.Y. (2018). Nanoparticle delivery of CRISPR into the brain rescues a mouse model of fragile X syndrome from exaggerated repetitive behaviours. Nat. Biomed. Eng. *2*, 497–507. https://doi.org/10.1038/s41551-018-0252-8.
20. Rock, J.M., Hopkins, F.F., Chavez, A., Diallo, M., Chase, M.R., Gerrick, E.R., Pritchard, J.R., Church, G.M., Rubin, E.J., Sassetti, C.M., et al. (2017). Programmable transcriptional repression in mycobacteria using an orthogonal CRISPR interference platform. Nat. Microbiol. *2*, 16274. https://doi.org/10.1038/nmicrobiol.2016.274.
21. Cui, L., and Bikard, D. (2016). Consequences of Cas9 cleavage in the chromosome of Escherichia coli. Nucleic Acids Res. *44*, 4243–4251. https://doi.org/10.1093/nar/gkw223.
22. Kelkar, A., Zhu, Y., Groth, T., Stolfa, G., Stablewski, A.B., Singhi, N., Nemeth, M., and Neelamegham, S. (2020). Doxycycline-Dependent Self-Inactivation of CRISPR-Cas9 to Temporally Regulate On- and Off-Target Editing. Mol. Ther. *28*, 29–41. https://doi.org/10.1016/j.ymthe.2019.09.006.
23. Lundin, A., Porritt, M.J., Jaiswal, H., Seeliger, F., Johansson, C., Bidar, A.W., Badertscher, L., Wimberger, S., Davies, E.J., Hardaker, E., et al. (2020). Development of an ObLiGaRe Doxycycline Inducible Cas9 system for pre-clinical cancer drug discovery. Nat. Commun. *11*, 4903. https://doi.org/10.1038/s41467-020-18548-9.
24. Dow, L.E., Fisher, J., O'Rourke, K.P., Muley, A., Kastenhuber, E.R., Livshits, G., Tschaharganeh, D.F., Socci, N.D., and Lowe, S.W. (2015). Inducible in vivo genome editing with CRISPR-Cas9. Nat. Biotechnol. *33*, 390–394. https://doi.org/10.1038/nbt.3155.
25. Lewis, K.T., Oles, L.R., and MacDougald, O.A. (2022). Tetracycline response element driven Cre causes ectopic recombinase activity independent of transactivator element. Mol. Metab. *61*, 101501. https://doi.org/10.1016/j.molmet.2022.101501.
26. Nguyen, D.P., Miyaoka, Y., Gilbert, L.A., Mayerl, S.J., Lee, B.H., Weissman, J.S., Conklin, B.R., and Wells, J.A. (2016). Ligand-binding domains of nuclear receptors facilitate tight control of split CRISPR activity. Nat. Commun. *7*, 12009. https://doi.org/10.1038/ncomms12009.
27. Mandegar, M.A., Huebsch, N., Frolov, E.B., Shin, E., Truong, A., Olvera, M.P., Chan, A.H., Miyaoka, Y., Holmes, K., Spencer, C.I., et al. (2016). CRISPR Interference Efficiently Induces Specific and Reversible Gene Silencing in Human iPSCs. Cell Stem Cell *18*, 541–553. https://doi.org/10.1016/j.stem.2016.01.022.
28. Nihongaki, Y., Otabe, T., Ueda, Y., and Sato, M. (2019). A split CRISPR-Cpf1 platform for inducible genome editing and gene activation. Nat. Chem. Biol. *15*, 882–888. https://doi.org/10.1038/s41589-019-0338-y.
29. Zetsche, B., Volz, S.E., and Zhang, F. (2015). A split-Cas9 architecture for inducible genome editing and transcription modulation. Nat. Biotechnol. *33*, 139–142. https://doi.org/10.1038/nbt.3149.
30. Nihongaki, Y., Kawano, F., Nakajima, T., and Sato, M. (2015). Photoactivatable CRISPR-Cas9 for optogenetic genome editing. Nat. Biotechnol. *33*, 755–760. https://doi.org/10.1038/nbt.3245.
31. Rotheneichner, P., Romanelli, P., Bieler, L., Pagitsch, S., Zaunmair, P., Kreutzer, C., König, R., Marschallinger, J., Aigner, L., and Couillard-Després, S. (2017). Tamoxifen Activation of Cre-Recombinase Has No Persisting Effects on Adult Neurogenesis or Learning and Anxiety. Front. Neurosci. *11*, 27. https://doi.org/10.3389/fnins.2017.00027.
32. Maji, B., Moore, C.L., Zetsche, B., Volz, S.E., Zhang, F., Shoulders, M.D., and Choudhary, A. (2017). Multidimensional chemical control of CRISPR-Cas9. Nat. Chem. Biol. *13*, 9–11. https://doi.org/10.1038/nchembio.2224.
33. Lundstrom, K. (2018). Viral Vectors in Gene Therapy. Diseases *6*, 42. https://doi.org/10.3390/diseases6020042.
34. Xu, C.L., Ruan, M.Z.C., Mahajan, V.B., and Tsang, S.H. (2019). Viral Delivery Systems for CRISPR. Viruses *11*, 28. https://doi.org/10.3390/v11010028.
35. Bulcha, J.T., Wang, Y., Ma, H., Tai, P.W.L., and Gao, G. (2021). Viral vector platforms within the gene therapy landscape. Signal Transduct. Target. Ther. *6*, 53. https://doi.org/10.1038/s41392-021-00487-6.
36. Rittiner, J.E., Moncalvo, M., Chiba-Falek, O., and Kantor, B. (2020). Gene-Editing Technologies Paired With Viral Vectors for Translational Research Into Neurodegenerative Diseases. Front. Mol. Neurosci. *13*, 148. https://doi.org/10.3389/fnmol.2020.00148.
37. Rauch, B.J., Silvis, M.R., Hultquist, J.F., Waters, C.S., McGregor, M.J., Krogan, N.J., and Bondy-Denomy, J. (2017). Inhibition of CRISPR-Cas9 with Bacteriophage Proteins. Cell *168*, 150–158.e10. https://doi.org/10.1016/j.cell.2016.12.009.
38. Pawluk, A., Amrani, N., Zhang, Y., Garcia, B., Hidalgo-Reyes, Y., Lee, J., Edraki, A., Shah, M., Sontheimer, E.J., Maxwell, K.L., and Davidson, A.R. (2016). Naturally Occurring Off-Switches for CRISPR-Cas9. Cell *167*, 1829–1838.e9. https://doi.org/10.1016/j.cell.2016.11.017.
39. Meacham, Z., de Tacca, L.A., Bondy-Denomy, J., Rabuka, D., and Schelle, M. (2023). Cas9 degradation in human cells using phage anti-CRISPR proteins. PLoS Biol. *21*, e3002431. https://doi.org/10.1371/journal.pbio.3002431.
40. Harrington, L.B., Doxzen, K.W., Ma, E., Liu, J.-J., Knott, G.J., Edraki, A., Garcia, B., Amrani, N., Chen, J.S., Cofsky, J.C., et al. (2017). A Broad-Spectrum Inhibitor of CRISPR-Cas9. Cell *170*, 1224–1233.e15. https://doi.org/10.1016/j.cell.2017.07.037.
41. Collins, G.A., and Goldberg, A.L. (2017). The Logic of the 26S Proteasome. Cell *169*, 792–806. https://doi.org/10.1016/j.cell.2017.04.023.
42. Okoye, C.N., Rowling, P.J.E., Itzhaki, L.S., and Lindon, C. (2022). Counting Degrons: Lessons From Multivalent Substrates for Targeted Protein Degradation. Front. Physiol. *13*, 913063. https://doi.org/10.3389/fphys.2022.913063.
43. Macdonald, L., Taylor, G., Brisbane, J., Christodoulou, E., Scott, L., Von Kriegsheim, A., Rossant, J., Gu, B., and Wood, A. (2022). Rapid and specific degradation of endogenous proteins in mouse models using auxin-inducible degrons. eLife *11*. https://doi.org/10.7554/eLife.77987.
44. Kleinjan, D.A., Wardrope, C., Nga Sou, S., and Rosser, S.J. (2017). Drug-tunable multidimensional synthetic gene control using inducible degron-tagged dCas9 effectors. Nat. Commun. *8*, 1191. https://doi.org/10.1038/s41467-017-01222-y.
45. Yesbolatova, A., Saito, Y., and Kanemaki, M.T. (2020). Constructing Auxin-Inducible Degron Mutants Using an All-in-One Vector. Pharmaceuticals *13*, 103. https://doi.org/10.3390/ph13050103.
46. Nabet, B., Roberts, J.M., Buckley, D.L., Paulk, J., Dastjerdi, S., Yang, A., Leggett, A. L., Erb, M.A., Lawlor, M.A., Souza, A., et al. (2018). The dTAG system for immediate and target-specific protein degradation. Nat. Chem. Biol. *14*, 431–441. https://doi.org/10.1038/s41589-018-0021-8.

47. Wu, Y., Yang, L., Chang, T., Kandeel, F., and Yee, J.-K. (2020). A Small Molecule-Controlled Cas9 Repressible System. Molecular therapy. Nucleic acids *19*, 922–932. https://doi.org/10.1016/j.omtn.2019.12.026.

48. Koduri, V., McBrayer, S.K., Liberzon, E., Wang, A.C., Briggs, K.J., Cho, H., and Kaelin, W.G., Jr. (2019). Peptidic degron for IMiD-induced degradation of heterologous proteins. Proc. Natl. Acad. Sci. USA *116*, 2539–2544. https://doi.org/10.1073/pnas.1818109116.

49. Zhu, Y.X., Braggio, E., Shi, C.-X., Bruins, L.A., Schmidt, J.E., Van Wier, S., Chang, X.-B., Bjorklund, C.C., Fonseca, R., Bergsagel, P.L., et al. (2011). Cereblon expression is required for the antimyeloma activity of lenalidomide and pomalidomide. Blood *118*, 4771–4779. https://doi.org/10.1182/blood-2011-05-356063.

50. Chamberlain, P.P., Lopez-Girona, A., Miller, K., Carmel, G., Pagarigan, B., Chie-Leon, B., Rychak, E., Corral, L.G., Ren, Y.J., Wang, M., et al. (2014). Structure of the human Cereblon-DDB1-lenalidomide complex reveals basis for responsiveness to thalidomide analogs. Nat. Struct. Mol. Biol. *21*, 803–809. https://doi.org/10.1038/nsmb.2874.

51. Brennan, P.J., Saunders, R.E., Spanou, M., Serafini, M., Sun, L., Heger, G.P., Konopacka, A., Beveridge, R.D., Gordon, L., Bunally, S.B., et al. (2024). Orthogonal IMiD-Degron Pairs Induce Selective Protein Degradation in Cells. Preprint at bioRxiv. https://doi.org/10.1101/2024.03.15.585309.

52. Takwale, A.D., Kim, E.Y., Jang, Y., Lee, D.H., Kim, S., Choi, Y., Kim, J.H., Lee, D.Y., Kim, Y., Lee, S.M., et al. (2022). Structure-activity relationship analysis of novel GSPT1 degraders based on benzotriazinone scaffold and its antitumor effect on xenograft mouse model. Bioorg. Chem. *127*, 105923. https://doi.org/10.1016/j.bioorg.2022.105923.

53. Chew, W.L., Tabebordbar, M., Cheng, J.K.W., Mali, P., Wu, E.Y., Ng, A.H.M., Zhu, K., Wagers, A.J., and Church, G.M. (2016). A multifunctional AAV-CRISPR-Cas9 and its host response. Nat. Methods *13*, 868–874. https://doi.org/10.1038/nmeth.3993.

54. Khajanchi, N., and Saha, K. (2022). Controlling CRISPR with small molecule regulation for somatic cell genome editing. Mol. Ther. *30*, 17–31. https://doi.org/10.1016/j.ymthe.2021.06.014.

55. Zhao, Y., and Huang, L. (2014). Lipid nanoparticles for gene delivery. Adv. Genet. *88*, 13–36. https://doi.org/10.1016/B978-0-12-800148-6.00002-X.

56. Im, S.H., Jang, M., Park, J.-H., and Chung, H.J. (2024). Finely tuned ionizable lipid nanoparticles for CRISPR/Cas9 ribonucleoprotein delivery and gene editing. J. Nanobiotechnol. *22*, 175. https://doi.org/10.1186/s12951-024-02427-2.

57. Walther, J., Porenta, D., Wilbie, D., Seinen, C., Benne, N., Yang, Q., de Jong, O.G., Lei, Z., and Mastrobattista, E. (2024). Comparative analysis of lipid Nanoparticle-Mediated delivery of CRISPR-Cas9 RNP versus mRNA/sgRNA for gene editing in vitro and in vivo. Eur. J. Pharm. Biopharm. *196*, 114207. https://doi.org/10.1016/j.ejpb.2024.114207.

58. Metzger, J.M., Wang, Y., Neuman, S.S., Snow, K.J., Murray, S.A., Lutz, C.M., Bondarenko, V., Felton, J., Gimse, K., Xie, R., et al. (2023). Efficient in vivo neuronal genome editing in the mouse brain using nanocapsules containing CRISPR-Cas9 ribonucleoproteins. Biomaterials *293*, 121959. https://doi.org/10.1016/j.biomaterials.2022.121959.

59. Chen, G., Abdeen, A.A., Wang, Y., Shahi, P.K., Robertson, S., Xie, R., Suzuki, M., Pattnaik, B.R., Saha, K., and Gong, S. (2019). A biodegradable nanocapsule delivers a Cas9 ribonucleoprotein complex for in vivo genome editing. Nat. Nanotechnol. *14*, 974–980. https://doi.org/10.1038/s41565-019-0539-2.

60. Mali, P., Yang, L., Esvelt, K.M., Aach, J., Guell, M., DiCarlo, J.E., Norville, J.E., and Church, G.M. (2013). RNA-guided human genome engineering via Cas9. Science *339*, 823–826. https://doi.org/10.1126/science.1232033.

61. Milone, M.C., and O'Doherty, U. (2018). Clinical use of lentiviral vectors. Leukemia *32*, 1529–1541. https://doi.org/10.1038/s41375-018-0106-0.

62. Eichler, F., Duncan, C., Musolino, P.L., Orchard, P.J., De Oliveira, S., Thrasher, A.J., Armant, M., Dansereau, C., Lund, T.C., Miller, W.P., et al. (2017). Hematopoietic Stem-Cell Gene Therapy for Cerebral Adrenoleukodystrophy. N. Engl. J. Med. *377*, 1630–1638. https://doi.org/10.1056/NEJMoa1700554.

63. Comisel, R.-M., Kara, B., Fiesser, F.H., and Farid, S.S. (2021). Lentiviral vector bioprocess economics for cell and gene therapy commercialization. Biochem. Eng. J. *167*, 107868. https://doi.org/10.1016/j.bej.2020.107868.

64. (2019). Gene therapy's next installment. Nat. Biotechnol. *37*, 697. https://doi.org/10.1038/s41587-019-0194-z.

65. Li, Y., Xu, Y., Liu, L., Wang, X., Palmisano, M., and Zhou, S. (2015). Population pharmacokinetics of pomalidomide. J. Clin. Pharmacol. *55*, 563–572. https://doi.org/10.1002/jcph.455.

66. Lazzarotto, C.R., Malinin, N.L., Li, Y., Zhang, R., Yang, Y., Lee, G., Cowley, E., He, Y., Lan, X., Jividen, K., et al. (2020). CHANGE-seq reveals genetic and epigenetic effects on CRISPR-Cas9 genome-wide activity. Nat. Biotechnol. *38*, 1317–1327. https://doi.org/10.1038/s41587-020-0555-7.

67. Chen, Y., Jiang, Y., Lao, J., Zhou, Y., Su, L., and Huang, X. (2022). Characterization and Functional Study of FAM49B Reveals Its Effect on Cell Proliferation in HEK293T Cells. Genes *13*, 388. https://doi.org/10.3390/genes13020388.

68. Li, J., Jing, Y., Liu, Y., Ru, Y., Ju, M., Zhao, Y., and Li, G. (2021). Large chromosomal deletions and impaired homologous recombination repairing in HEK293T cells exposed to polychlorinated biphenyl 153. PeerJ *9*, e11816. https://doi.org/10.7717/peerj.11816.

69. Malm, M., Saghaleyni, R., Lundqvist, M., Giudici, M., Chotteau, V., Field, R., Varley, P.G., Hatton, D., Grassi, L., Svensson, T., et al. (2020). Evolution from adherent to suspension: systems biology of HEK293 cell line development. Sci. Rep. *10*, 18996. https://doi.org/10.1038/s41598-020-76137-8.

70. Michiko, M., Akira, W., Yasuo, K., Yasuhiko, H., Chikako, M., Takashi, D., Masashi, F., Hiroshi, A., Sakai, N., Yumiko, S., et al. (2017). Autologous Induced Stem-Cell–Derived Retinal Cells for Macular Degeneration. N. Engl. J. Med. *376*, 1038–1046. https://doi.org/10.1056/NEJMoa1608368.

71. Shi, Y., Inoue, H., Wu, J.C., and Yamanaka, S. (2017). Induced pluripotent stem cell technology: a decade of progress. Nat. Rev. Drug Discov. *16*, 115–130. https://doi.org/10.1038/nrd.2016.245.

72. Molugu, K., Khajanchi, N., Lazzarotto, C.R., Tsai, S.Q., and Saha, K. (2023). Trichostatin A for Efficient CRISPR-Cas9 Gene Editing of Human Pluripotent Stem Cells. CRISPR J. *6*, 473–485. https://doi.org/10.1089/crispr.2023.0033.

73. Hohenstein, K.A., Pyle, A.D., Chern, J.Y., Lock, L.F., and Donovan, P.J. (2008). Nucleofection mediates high-efficiency stable gene knockdown and transgene expression in human embryonic stem cells. Stem Cell *26*, 1436–1443. https://doi.org/10.1634/stemcells.2007-0857.

74. Huston, N.C., Tycko, J., Tillotson, E.L., Wilson, C.J., Myer, V.E., Jayaram, H., and Steinberg, B.E. (2019). Identification of Guide-Intrinsic Determinants of Cas9 Specificity. CRISPR J. *2*, 172–185. https://doi.org/10.1089/crispr.2019.0009.

75. Tsai, S.Q., Zheng, Z., Nguyen, N.T., Liebers, M., Topkar, V.V., Thapar, V., Wyvekens, N., Khayter, C., Iafrate, A.J., Le, L.P., et al. (2015). GUIDE-seq enables genome-wide profiling of off-target cleavage by CRISPR-Cas nucleases. Nat. Biotechnol. *33*, 187–197. https://doi.org/10.1038/nbt.3117.

76. Baruteau, J., Brunetti-Pierri, N., and Gissen, P. (2024). Liver-directed gene therapy for inherited metabolic diseases. J. Inherit. Metab. Dis. *47*, 9–21. https://doi.org/10.1002/jimd.12709.

77. Cozmescu, A.C., Counsell, J., and Gissen, P. (2021). Gene therapies targeting the liver. J. Hepatol. *74*, 235–236. https://doi.org/10.1016/j.jhep.2020.08.003.

78. Zhang, K., Wan, P., Wang, L., Wang, Z., Tan, F., Li, J., Ma, X., Cen, J., Yuan, X., Liu, Y., et al. (2024). Efficient expansion and CRISPR-Cas9-mediated gene correction of patient-derived hepatocytes for treatment of inherited liver diseases. Cell Stem Cell *31*, 1187–1202.e8. https://doi.org/10.1016/j.stem.2024.04.022.

79. Musunuru, K., Chadwick, A.C., Mizoguchi, T., Garcia, S.P., DeNizio, J.E., Reiss, C. W., Wang, K., Iyer, S., Dutta, C., Clendaniel, V., et al. (2021). In vivo CRISPR base editing of PCSK9 durably lowers cholesterol in primates. Nature *593*, 429–434. https://doi.org/10.1038/s41586-021-03534-y.

80. Chadwick, A.C., Wang, X., and Musunuru, K. (2017). In Vivo Base Editing of PCSK9 (Proprotein Convertase Subtilisin/Kexin Type 9) as a Therapeutic Alternative to Genome Editing. Arterioscler. Thromb. Vasc. Biol. *37*, 1741–1747. https://doi.org/10.1161/ATVBAHA.117.309881.

81. Rothgangl, T., Dennis, M.K., Lin, P.J.C., Oka, R., Witzigmann, D., Villiger, L., Qi, W., Hruzova, M., Kissling, L., Lenggenhager, D., et al. (2021). In vivo adenine base editing of PCSK9 in macaques reduces LDL cholesterol levels. Nat. Biotechnol. *39*, 949–957. https://doi.org/10.1038/s41587-021-00933-4.

82. Carreras, A., Pane, L.S., Nitsch, R., Madeyski-Bengtson, K., Porritt, M., Akcakaya, P., Taheri-Ghahfarokhi, A., Ericson, E., Bjursell, M., Perez-Alcazar, M., et al. (2019). In vivo genome and base editing of a human PCSK9 knock-in hypercholesterolemic mouse model. BMC Biol. *17*, 4. https://doi.org/10.1186/s12915-018-0624-2.

83. Sun, J., Carlson-Stevermer, J., Das, U., Shen, M., Delenclos, M., Snead, A.M., Koo, S.Y., Wang, L., Qiao, D., Loi, J., et al. (2019). CRISPR/Cas9 editing of APP C-terminus attenuates β-cleavage and promotes α-cleavage. Nat. Commun. *10*, 53. https://doi.org/10.1038/s41467-018-07971-8.

84. Kim, S., Kim, D., Cho, S.W., Kim, J., and Kim, J.-S. (2014). Highly efficient RNA-guided genome editing in human cells via delivery of purified Cas9 ribonucleoproteins. Genome Res. *24*, 1012–1019. https://doi.org/10.1101/gr.171322.113.

85. Hershko, A. (2005). The ubiquitin system for protein degradation and some of its roles in the control of the cell division cycle. Cell Death Differ. *12*, 1191–1197. https://doi.org/10.1038/sj.cdd.4401702.

86. Sperling, A.S., Burgess, M., Keshishian, H., Gasser, J.A., Bhatt, S., Jan, M., Słabicki, M., Sellar, R.S., Fink, E.C., Miller, P.G., et al. (2019). Patterns of substrate affinity, competition, and degradation kinetics underlie biological activity of thalidomide analogs. Blood *134*, 160–170. https://doi.org/10.1182/blood.2019000789.

87. Kowalski, T.W., Feira, M.F., Lord, V.O., Gomes, J.d.A., Giudicelli, G.C., Fraga, L.R., Sanseverino, M.T.V., Recamonde-Mendoza, M., Schuler-Faccini, L., and Vianna, F.S. L. (2023). A New Strategy for the Old Challenge of Thalidomide: Systems Biology Prioritization of Potential Immunomodulatory Drug (IMiD)-Targeted Transcription Factors. Int. J. Mol. Sci. *24*, 11515. https://doi.org/10.3390/ijms241411515.

88. Gao, S., Wang, S., and Song, Y. (2020). Novel immunomodulatory drugs and neo-substrates. Biomark. Res. *8*, 2. https://doi.org/10.1186/s40364-020-0182-y.

89. Sievers, Q.L., Petzold, G., Bunker, R.D., Renneville, A., Słabicki, M., Liddicoat, B.J., Abdulrahman, W., Mikkelsen, T., Ebert, B.L., and Thomä, N.H. (2018). Defining the human C2H2 zinc finger degrome targeted by thalidomide analogs through CRBN. Science *362*, eaat0572. https://doi.org/10.1126/science.aat0572.

90. Shen, C., Nayak, A., Neitzel, L.R., Adams, A.A., Silver-Isenstadt, M., Sawyer, L.M., Benchabane, H., Wang, H., Bunnag, N., Li, B., et al. (2021). The E3 ubiquitin ligase component, Cereblon, is an evolutionarily conserved regulator of Wnt signaling. Nat. Commun. *12*, 5263. https://doi.org/10.1038/s41467-021-25634-z.

91. Yamamoto, J., Suwa, T., Murase, Y., Tateno, S., Mizutome, H., Asatsuma-Okumura, T., Shimizu, N., Kishi, T., Momose, S., Kizaki, M., et al. (2020). ARID2 is a pomalidomide-dependent CRL4CRBN substrate in multiple myeloma cells. Nat. Chem. Biol. *16*, 1208–1217. https://doi.org/10.1038/s41589-020-0645-3.

92. Bjorklund, C.C., Lu, L., Kang, J., Hagner, P.R., Havens, C.G., Amatangelo, M., Wang, M., Ren, Y., Couto, S., Breider, M., et al. (2015). Rate of CRL4(CRBN) substrate Ikaros and Aiolos degradation underlies differential activity of lenalidomide and pomalidomide in multiple myeloma cells by regulation of c-Myc and IRF4. Blood Cancer J. *5*, e354. https://doi.org/10.1038/bcj.2015.66.

93. Donovan, K.A., An, J., Nowak, R.P., Yuan, J.C., Fink, E.C., Berry, B.C., Ebert, B.L., and Fischer, E.S. (2018). Thalidomide promotes degradation of SALL4, a transcription factor implicated in Duane Radial Ray syndrome. eLife *7*, e38430. https://doi.org/10.7554/eLife.38430.

94. Matyskiela, M.E., Couto, S., Zheng, X., Lu, G., Hui, J., Stamp, K., Drew, C., Ren, Y., Wang, M., Carpenter, A., et al. (2018). SALL4 mediates teratogenicity as a thalidomide-dependent cereblon substrate. Nat. Chem. Biol. *14*, 981–987. https://doi.org/10.1038/s41589-018-0129-x.

95. Uhlen, M., Oksvold, P., Fagerberg, L., Lundberg, E., Jonasson, K., Forsberg, M., Zwahlen, M., Kampf, C., Wester, K., Hober, S., et al. (2010). Towards a knowledge-based Human Protein Atlas. Nat. Biotechnol. *28*, 1248–1250. https://doi.org/10.1038/nbt1210-1248.

96. (2024). The Human Protein Atlas. https://www.proteinatlas.org/.

97. Choi, E.H., Suh, S., Sears, A.E., Hołubowicz, R., Kedhar, S.R., Browne, A.W., and Palczewski, K. (2023). Genome editing in the treatment of ocular diseases. Exp. Mol. Med. *55*, 1678–1690. https://doi.org/10.1038/s12276-023-01057-2.

98. Mercer, J.A.M., DeCarlo, S.J., Roy Burman, S.S., Sreekanth, V., Nelson, A.T., Hunkeler, M., Chen, P.J., Donovan, K.A., Kokkonda, P., Tiwari, P.K., et al. (2024). Continuous evolution of compact protein degradation tags regulated by selective molecular glues. Science *383*, eadk4422. https://doi.org/10.1126/science.adk4422.

99. Fouquet, G., Bories, C., Guidez, S., Renaud, L., Herbaux, C., Javed, S., Facon, T., and Leleu, X. (2014). Pomalidomide for multiple myeloma. Expert Rev. Hematol. *7*, 719–731. https://doi.org/10.1586/17474086.2014.966074.

100. Lurain, K., Polizzotto, M.N., Krug, L.T., Shoemaker, G., Singh, A., Jensen, S.M.R., Wyvill, K.M., Ramaswami, R., Uldrick, T.S., Yarchoan, R., and Sereti, I. (2023). Immunophenotypic analysis in participants with Kaposi sarcoma following pomalidomide administration. AIDS *37*, 1693–1703. https://doi.org/10.1097/QAD.0000000000003627.

101. Flury, A., Aljayousi, L., Park, H.-J., Khakpour, M., Mechler, J., Aziz, S., McGrath, J. D., Deme, P., Sandberg, C., González Ibánez, F., et al. (2025). A neurogenerative cellular stress response linked to dark microglia and toxic lipid secretion. Neuron. *113*, 554–571.e14. https://doi.org/10.1016/j.neuron.2024.11.018.

102. Aulston, B.D., Gimse, K., Bazick, H.O., Kramar, E.A., Pizzo, D.P., Parra-Rivas, L.A., Sun, J., Branes-Guerrero, K., Checka, N., Bagheri, N., et al. (2025). Long term rescue of Alzheimer's deficits in vivo by one-time gene-editing of App C-terminus. Preprint at bioRxiv. https://doi.org/10.1101/2024.06.08.598099.

103. Fu, Y.-W., Dai, X.-Y., Wang, W.-T., Yang, Z.-X., Zhao, J.-J., Zhang, J.-P., Wen, W., Zhang, F., Oberg, K.C., Zhang, L., et al. (2021). Dynamics and competition of CRISPR-Cas9 ribonucleoproteins and AAV donor-mediated NHEJ, MMEJ and HDR editing. Nucleic Acids Res. *49*, 969–985. https://doi.org/10.1093/nar/gkaa1251.

104. McKinnon, P.J. (2013). Maintaining genome stability in the nervous system. Nat. Neurosci. *16*, 1523–1529. https://doi.org/10.1038/nn.3537.

105. Monteys, A.M., Hundley, A.A., Ranum, P.T., Tecedor, L., Muehlmatt, A., Lim, E., Lukashev, D., Sivasankaran, R., and Davidson, B.L. (2021). Regulated control of gene therapies by drug-induced splicing. Nature *596*, 291–295. https://doi.org/10.1038/s41586-021-03770-2.

106. Sreekanth, V., Jan, M., Zhao, K.T., Lim, D., Davis, J.R., McConkey, M., Kovalcik, V., Barkal, S., Law, B.K., Fife, J., et al. (2023). A molecular glue approach to control the half-life of CRISPR-based technologies. Preprint at bioRxiv. https://doi.org/10.1101/2023.03.12.531757.

107. Jackson, K.L., Dayton, R.D., Deverman, B.E., and Klein, R.L. (2016). Better targeting, better efficiency for wide-scale neuronal transduction with the synapsin promoter and AAV-PHP. Front. Mol. Neurosci. *9*, 116. https://doi.org/10.3389/fnmol.2016.00116.

108. Gurumoorthy, N., Nordin, F., Tye, G.J., Wan Kamarul Zaman, W.S., Ng, M.H., and Ng, M.H. (2022). Non-integrating Lentiviral vectors in clinical applications: A glance through. Biomedicines *10*, 107. https://doi.org/10.3390/biomedicines10010107.

109. Chen, C., Ma, Y., Du, S., Wu, Y., Shen, P., Yan, T., Li, X., Song, Y., Zha, Z., and Han, X. (2021). Controlled CRISPR-Cas9 Ribonucleoprotein Delivery for Sensitized Photothermal Therapy. Small *17*, e2101155. https://doi.org/10.1002/smll.202101155.

110. Pan, Y., Yang, J., Luan, X., Liu, X., Li, X., Yang, J., Huang, T., Sun, L., Wang, Y., Lin, Y., and Song, Y. (2019). Near-infrared upconversion-activated CRISPR-Cas9 system: A remote-controlled gene editing platform. Sci. Adv. *5*, eaav7199. https://doi.org/10.1126/sciadv.aav7199.

111. Deng, S., Li, X., Liu, S., Chen, J., Li, M., Chew, S.Y., Leong, K.W., and Cheng, D. (2020). Codelivery of CRISPR-Cas9 and chlorin e6 for spatially controlled tumor-specific gene editing with synergistic drug effects. Sci. Adv. *6*, eabb4005. https://doi.org/10.1126/sciadv.abb4005.

112. Lyu, Y., He, S., Li, J., Jiang, Y., Sun, H., Miao, Y., and Pu, K. (2019). A Photolabile Semiconducting Polymer Nanotransducer for Near-Infrared Regulation of CRISPR/Cas9 Gene Editing. Angew. Chem. *58*, 18197–18201. https://doi.org/10.1002/anie.201909264.

113. Yin, H., Sun, L., Pu, Y., Yu, J., Feng, W., Dong, C., Zhou, B., Du, D., Zhang, Y., Chen, Y., and Xu, H. (2021). Ultrasound-Controlled CRISPR/Cas9 System Augments Sonodynamic Therapy of Hepatocellular Carcinoma. ACS Cent. Sci. *7*, 2049–2062. https://doi.org/10.1021/acscentsci.1c01143.

114. Xie, R., Wang, X., Wang, Y., Ye, M., Zhao, Y., Yandell, B.S., and Gong, S. (2022). pH-Responsive Polymer Nanoparticles for Efficient Delivery of Cas9 Ribonucleoprotein With or Without Donor DNA. Adv. Mater. *34*, e2110618. https://doi.org/10.1002/adma.202110618.

115. Wang, Y., Shahi, P.K., Wang, X., Xie, R., Zhao, Y., Wu, M., Roge, S., Pattnaik, B.R., and Gong, S. (2021). In vivo targeted delivery of nucleic acids and CRISPR genome editors enabled by GSH-responsive silica nanoparticles. J. Control. Release *336*, 296–309. https://doi.org/10.1016/j.jconrel.2021.06.030.

116. Zou, Y., Sun, X., Yang, Q., Zheng, M., Shimoni, O., Ruan, W., Wang, Y., Zhang, D., Yin, J., Huang, X., et al. (2022). Blood-brain barrier-penetrating single CRISPR-Cas9 nanocapsules for effective and safe glioblastoma gene therapy. Sci. Adv. *8*, eabm8011. https://doi.org/10.1126/sciadv.abm8011.

117. Peng, H., Le, C., Wu, J., Li, X.-F., Zhang, H., and Le, X.C. (2020). A Genome-Editing Nanomachine Constructed with a Clustered Regularly Interspaced Short Palindromic Repeats System and Activated by Near-Infrared Illumination. ACS Nano *14*, 2817–2826. https://doi.org/10.1021/acsnano.9b05276.

118. Moroz-Omori, E.V., Satyapertiwi, D., Ramel, M.-C., Høgset, H., Sunyovszki, I.K., Liu, Z., Wojciechowski, J.P., Zhang, Y., Grigsby, C.L., Brito, L., et al. (2020). Photoswitchable gRNAs for Spatiotemporally Controlled CRISPR-Cas-Based Genomic Regulation. ACS Cent. Sci. *6*, 695–703. https://doi.org/10.1021/acscentsci.9b01093.

119. Friedland, A.E., Baral, R., Singhal, P., Loveluck, K., Shen, S., Sanchez, M., Marco, E., Gotta, G.M., Maeder, M.L., Kennedy, E.M., et al. (2015). Characterization of Staphylococcus aureus Cas9: a smaller Cas9 for all-in-one adeno-associated virus delivery and paired nickase applications. Genome Biol. *16*, 257. https://doi.org/10.1186/s13059-015-0817-8.

120. Ibraheim, R., Song, C.-Q., Mir, A., Amrani, N., Xue, W., and Sontheimer, E.J. (2018). All-in-one adeno-associated virus delivery and genome editing by Neisseria meningitidis Cas9 in vivo. Genome Biol. *19*, 137. https://doi.org/10.1186/s13059-018-1515-0.

121. Lee, C.M., Cradick, T.J., and Bao, G. (2016). The Neisseria meningitidis CRISPR-Cas9 system enables specific genome editing in mammalian cells. Mol. Ther. *24*, 645–654. https://doi.org/10.1038/mt.2016.8.

122. Laurent, M., Geoffroy, M., Pavani, G., and Guiraud, S. (2024). CRISPR-based gene therapies: From preclinical to clinical treatments. Cells *13*, 800. https://doi.org/10.3390/cells13100800.

123. Lei, L., Pan, W., Shou, X., Shao, Y., Ye, S., Zhang, J., Kolliputi, N., and Shi, L. (2024). Nanomaterials-assisted gene editing and synthetic biology for optimizing the treatment of pulmonary diseases. J. Nanobiotechnol. *22*, 343. https://doi.org/10.1186/s12951-024-02627-w.

124. Jeong, S.H., Lee, H.J., and Lee, S.J. (2023). Recent advances in CRISPR-Cas technologies for synthetic biology. J. Microbiol. *61*, 13–36. https://doi.org/10.1007/s12275-022-00005-5.

125. (2025). Roadmap to Reducing Animal Testing in Preclinical Safety Studies.. https://www.fda.gov/media/186092/download?attachment.

126. Carbonneau, S., Sharma, S., Peng, L., Rajan, V., Hainzl, D., Henault, M., Yang, C., Hale, J., Shulok, J., Tallarico, J., et al. (2021). An IMiD-inducible degron provides reversible regulation for chimeric antigen receptor expression and activity. Cell Chem. Biol. *28*, 802–812.e6. https://doi.org/10.1016/j.chembiol.2020.11.012.

127. Jan, M., Scarfò, I., Larson, R.C., Walker, A., Schmidts, A., Guirguis, A.A., Gasser, J. A., Słabicki, M., Bouffard, A.A., Castano, A.P., et al. (2021). Reversible ON- and OFF-switch chimeric antigen receptors controlled by lenalidomide. Sci. Transl. Med. *13*, eabb6295. https://doi.org/10.1126/scitranslmed.abb6295.

128. Snetkova, V., Galan, C., Lopez, R., Rios, A.R., Kudo, T., Dorighi, K., Warming, S., and Haley, B.J. (2024). Degron-modified Cas12a enhances single-cell CRISPR screening. Preprint at bioRxiv. https://doi.org/10.1101/2024.12.08.627374.

129. Cong, L., Ran, F.A., Cox, D., Lin, S., Barretto, R., Habib, N., Hsu, P.D., Wu, X., Jiang, W., Marraffini, L.A., and Zhang, F. (2013). Multiplex genome engineering using CRISPR/Cas systems. Science *339*, 819–823. https://doi.org/10.1126/science.1231143.

130. Murty, T., and Mackall, C.L. (2021). Gene editing to enhance the efficacy of cancer cell therapies. Mol. Ther. *29*, 3153–3162. https://doi.org/10.1016/j.ymthe.2021.10.001.

131. Uddin, F., Rudin, C.M., and Sen, T. (2020). CRISPR gene therapy: Applications, limitations, and implications for the future. Front. Oncol. *10*, 1387. https://doi.org/10.3389/fonc.2020.01387.

132. Urnov, F.D., Miller, J.C., Lee, Y.-L., Beausejour, C.M., Rock, J.M., Augustus, S., Jamieson, A.C., Porteus, M.H., Gregory, P.D., and Holmes, M.C. (2005). Highly efficient endogenous human gene correction using designed zinc-finger nucleases. Nature *435*, 646–651. https://doi.org/10.1038/nature03556.

133. Joung, J.K., and Sander, J.D. (2013). TALENs: a widely applicable technology for targeted genome editing. Nat. Rev. Mol. Cell Biol. *14*, 49–55. https://doi.org/10.1038/nrm3486.

134. McCutcheon, S.R., Rohm, D., Iglesias, N., and Gersbach, C.A. (2024). Epigenome editing technologies for discovery and medicine. Nat. Biotechnol. *42*, 1199–1217. https://doi.org/10.1038/s41587-024-02320-1.

135. Tang, L. (2024). Epigenetic editing with CHARM. Nat. Methods *21*, 1410. https://doi.org/10.1038/s41592-024-02387-3.

136. Lampe, G.D., King, R.T., Halpin-Healy, T.S., Klompe, S.E., Hogan, M.I., Vo, P.L.H., Tang, S., Chavez, A., and Sternberg, S.H. (2024). Targeted DNA integration in human cells without double-strand breaks using CRISPR-associated transposases. Nat. Biotechnol. *42*, 87–98. https://doi.org/10.1038/s41587-023-01748-1.

137. Schmitt-Ulms, C., Kayabolen, A., Manero-Carranza, M., Zhou, N., Donnelly, K., Nuccio, S.P., Kato, K., Nishimasu, H., Gootenberg, J.S., and Abudayyeh, O.O. (2024). Programmable RNA writing with trans-splicing. Preprint at bioRxiv. https://doi.org/10.1101/2024.01.31.578223.

138. Villiger, L., Joung, J., Koblan, L., Weissman, J., Abudayyeh, O.O., and Gootenberg, J.S. (2024). CRISPR technologies for genome, epigenome and transcriptome editing. Nat. Rev. Mol. Cell Biol. *25*, 464–487. https://doi.org/10.1038/s41580-023-00697-6.

139. (2024). Cortical GABAergic Neurons. (BrainXell) https://brainxell.com/cortical-gabaergic-neurons.

140. Riss, T.L., Moravec, R.A., Niles, A.L., Duellman, S., Benink, H.A., Worzella, T.J., and Minor, L. (2016). Cell Viability Assays (Eli Lilly & Company and the National Center for Advancing Translational Sciences). https://www.ncbi.nlm.nih.gov/books/NBK144065/.

141. Pillai-Kastoori, L., Schutz-Geschwender, A.R., and Harford, J.A. (2020). A systematic approach to quantitative Western blot analysis. Anal. Biochem. *593*, 113608. https://doi.org/10.1016/j.ab.2020.113608.

142. Kabra, M., Shahi, P.K., Wang, Y., Sinha, D., Spillane, A., Newby, G.A., Saxena, S., Tong, Y., Chang, Y., Abdeen, A.A., et al. (2023). Nonviral base editing of KCNJ13 mutation preserves vision in a model of inherited retinal channelopathy. J. Clin. Investig. *133*, e171356. https://doi.org/10.1172/JCI171356.

OMTN, Volume *36*

Supplemental information

Controlling CRISPR-Cas9 genome editing in human cells using a molecular glue degrader

Namita Khajanchi, Vrusha Patel, Ronak Dua, Meha Kabra, Bikash R. Pattnaik, and Krishanu Saha

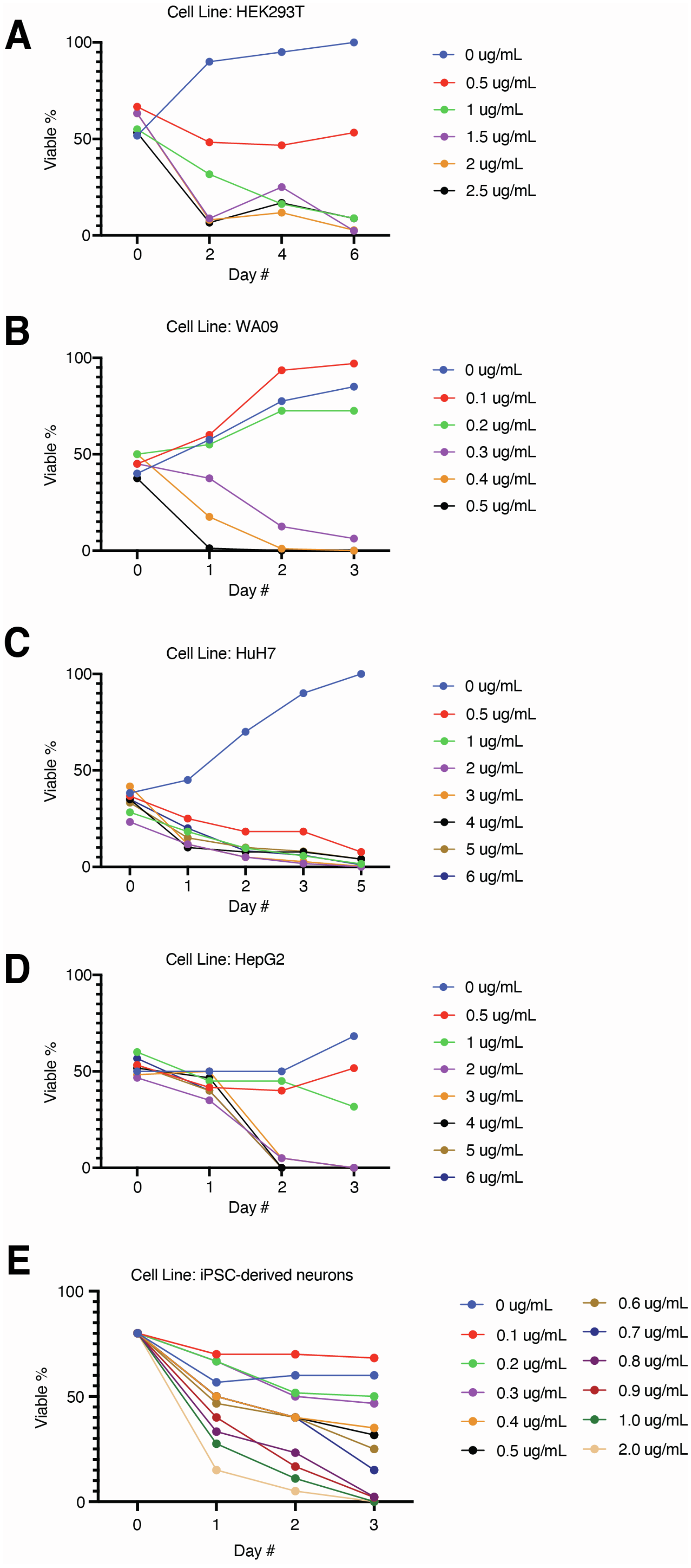
A
Cell Line: HEK293T
Viable %
Day #
0 ug/mL
0.5 ug/mL
1 ug/mL
1.5 ug/mL
2 ug/mL
2.5 ug/mL
B
Cell Line: WA09
Viable %
Day #
0 ug/mL
0.1 ug/mL
0.2 ug/mL
0.3 ug/mL
0.4 ug/mL
0.5 ug/mL
C
Cell Line: HuH7
Viable %
Day #
0 ug/mL
0.5 ug/mL
1 ug/mL
2 ug/mL
3 ug/mL
4 ug/mL
5 ug/mL
6 ug/mL
D
Cell Line: HepG2
Viable %
Day #
0 ug/mL
0.5 ug/mL
1 ug/mL
2 ug/mL
3 ug/mL
4 ug/mL
5 ug/mL
6 ug/mL
E
Cell Line: iPSC-derived neurons
Viable %
Day #
0 ug/mL
0.1 ug/mL
0.2 ug/mL
0.3 ug/mL
0.4 ug/mL
0.5 ug/mL
0.6 ug/mL
0.7 ug/mL
0.8 ug/mL
0.9 ug/mL
1.0 ug/mL
2.0 ug/mL

Figure S1. Puromycin (Puro) Kill Curves for **(A)** HEK293T, **(B)** WA09, **(C)** HuH7, **(D)** HepG2 cells, and **(E)** hiPSC-derived neurons. Cells were seeded at approximately 50% in all wells prior to starting the kill curve. **(A)** HEK cells were killed within 2 days with 1.5 - 2.5 µg/mL of puro. The lowest dose, 1.5 µg/mL puro was used. **(B)** WA09 were killed within a day with 0.5 µg/mL of puro and within 2 days with 0.4 µg/mL of puro. Because iPSCs are puromycin sensitive, 0.3 µg/mL puro was used. **(C)** HuH7 cells were killed within 5 days with 1 µg/mL - 6 µg/mL of puro. One of the lowest doses, 2 µg/mL puro was used to match the amount that is used in **(D)** HepG2 cells as both cells would be cultured at the same time. **(E)** **h**iPSC-neurons were killed within 3 days at puromycin concentrations of 0.8 µg/mL and higher. Since these neurons are sensitive, we used a lower dose of 0.7 µg/mL of puro. Data represented in line plots are means, n = 3 technical replicates per condition.

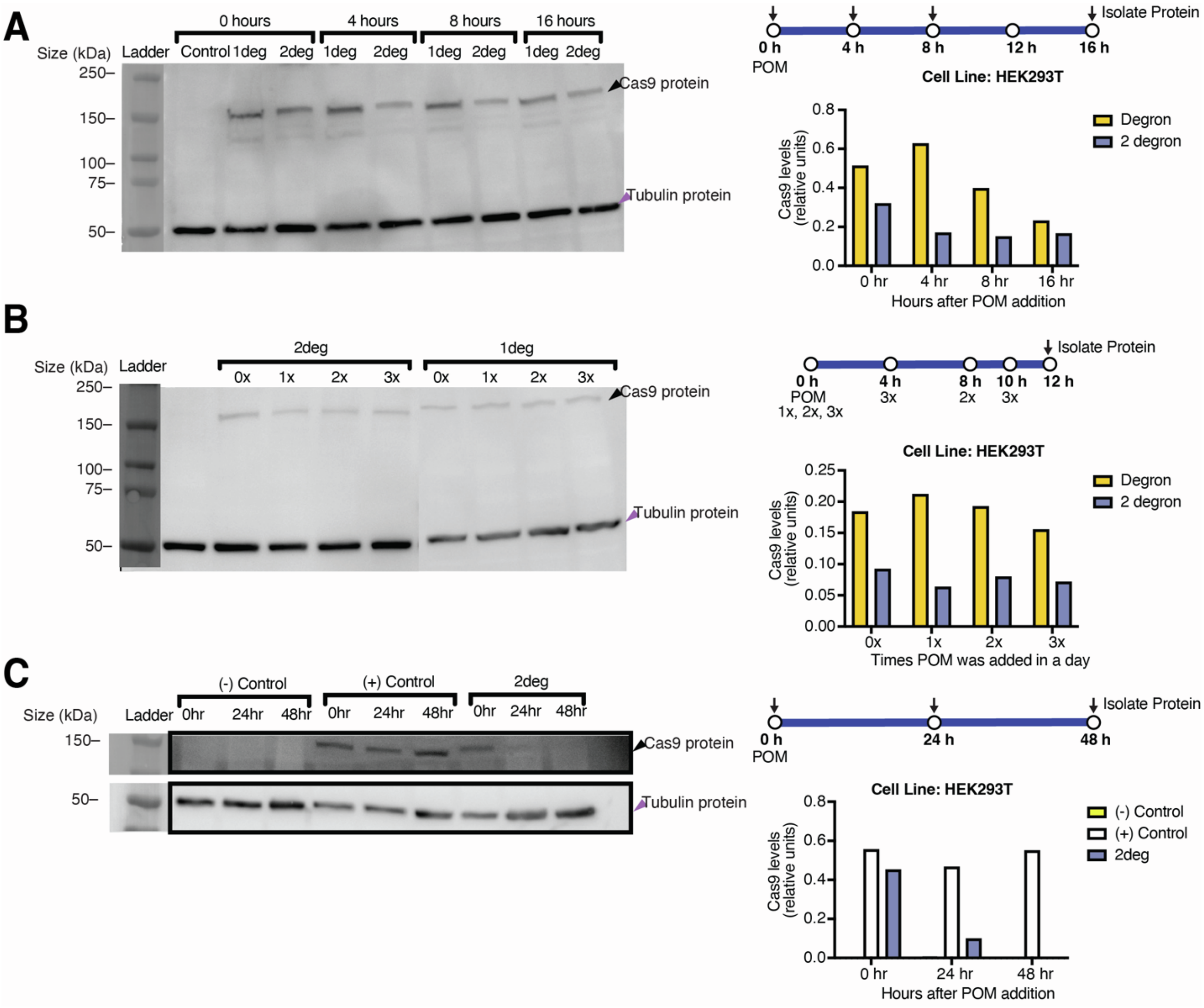

Figure S2. Western blots to determine degron construct to use. (**A**) Representative western blot for Cas9-1d and Cas9-d protein levels over the course of 16 hours after one dose of POM at hour 0. The half-life for Cas9-1d was 8.29 hours and for Cas9-d was 7.31 hours. Data represented in bar graphs are n = 1 sample. **(B)** Representative western blot showing Cas9 levels in cells after 24 hours when POM was added once, twice, or three times a day. There is no significant change in Cas9 levels. Data represented in bar graphs are n = 1 sample. (**C**) Representative western blot depicting untransduced cells (- Control), Cas9-mCherry (+ Control) and Cas9-d cells. POM was added to all cells at hour 0, and samples were isolated at 0, 24, and 48 hours. Data represented in bar graphs are n = 1 sample.

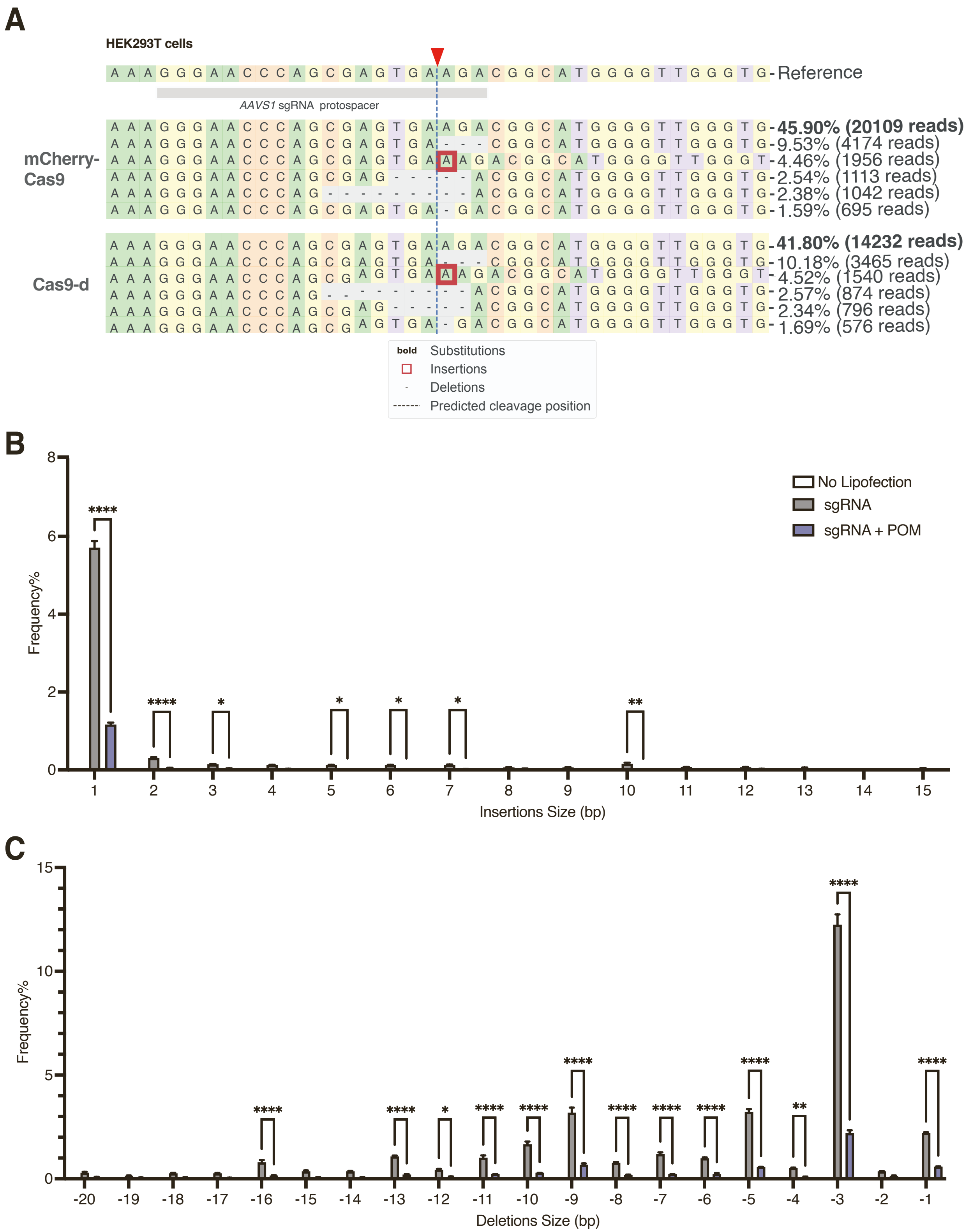

Figure S3. Decrease in indels in POM-induced Cas9-d HEK293T cells. Indel profiles after lipofection for **(A)** WT Cas9 (Cas9-mCherry) and Cas9-d at the *AAVS1* locus in HEK cells. The dotted line represents where the cut site is. Both indel profiles are similar except for extra inserts

in WT Cas9 cells. **(B)** The insertion spectrum at the AAVS1 on-target locus shows that there is an average of 4.9-fold change in frequency between significant sizes of insertions in POM-induced Cas9-d cells and Cas9-d cells. **(C)** The deletion spectrum at the AAVS1 on-target locus shows that there is an average of 9.1-fold change in frequency between significant sizes of deletions in POM-induced Cas9-d cells and Cas9-d cells. Data represented in bar graphs in (B) and (C) are represented as mean +/- SEM, n = 3 technical replicates per condition, p-values generated by two-way ANOVA; ns = $p \geq 0.05$ (not shown), * for $p < 0.05$, ** for $p < 0.01$, *** for $p < 0.001$, **** for $p < 0.0001$.

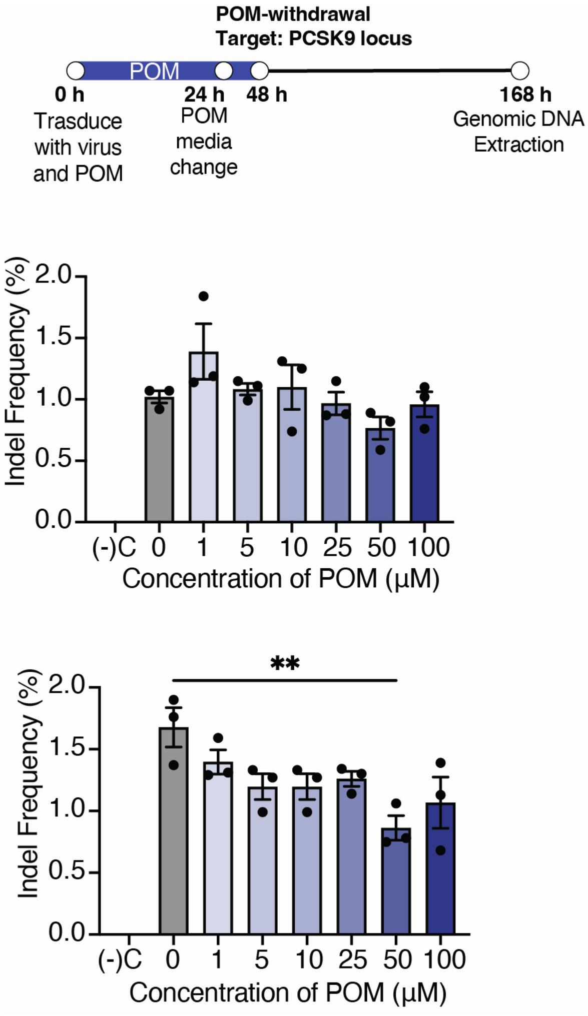

Figure S4. Hepatocyte withdrawal experiments.
On-target indel frequencies after 5 days of POM withdrawal within (top) HepG2 and (bottom) Huh7 cells. Editing increases upon POM at each concentration up to 2-fold when compared with POM-induced counterparts. Data represented in all bar graphs are represented as mean +/- SEM, n = 3 technical replicates per condition, p-values generated by two-way ANOVA; ns = $p \geq 0.05$ (not shown), * for $p < 0.05$, ** for $p < 0.01$, *** for $p < 0.001$, **** for $p < 0.0001$.

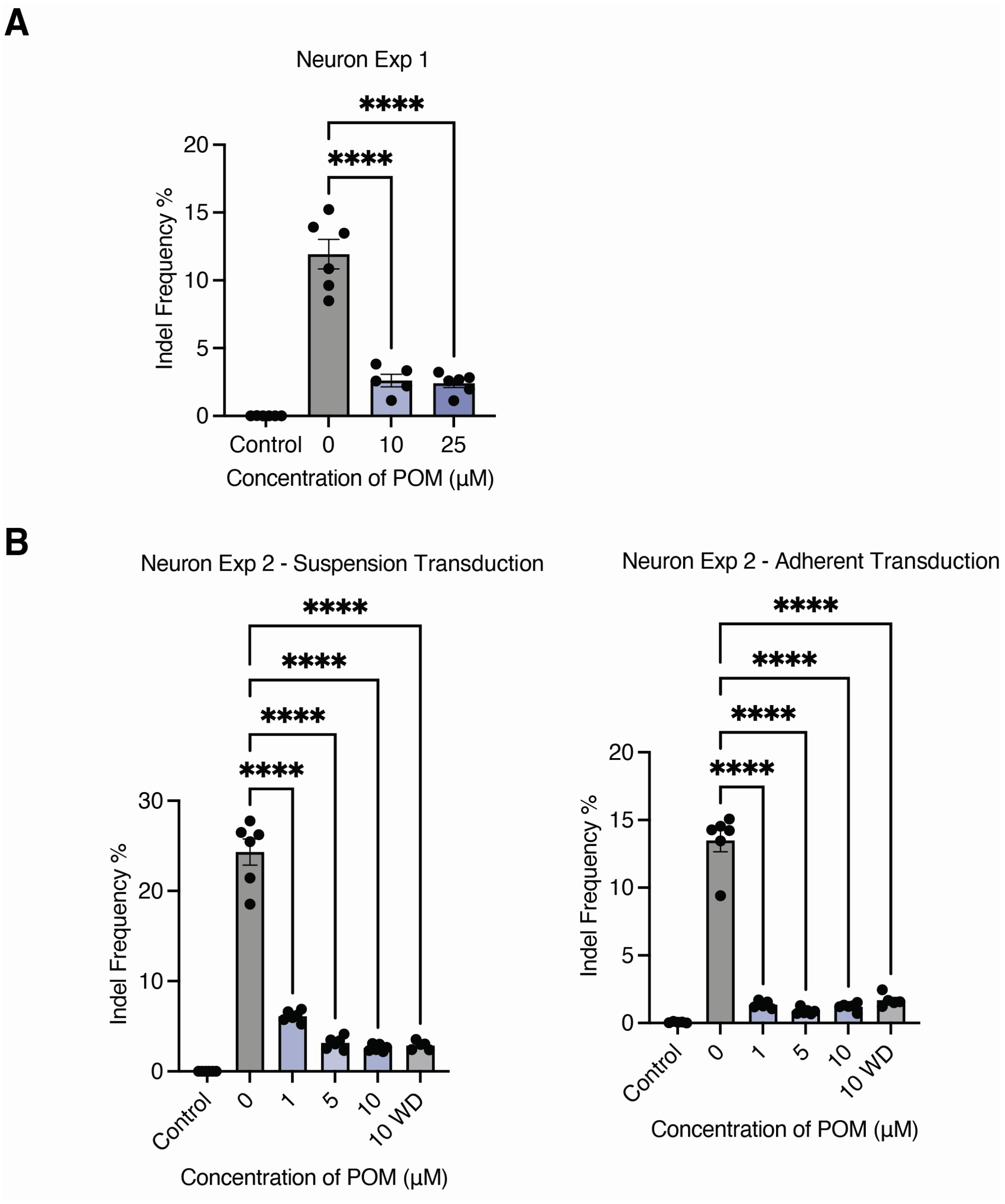

Figure S5. hiPSC-derived neuron replicates experiments.
(A) Neurons were transduced with Cas9-d virus while in suspension with POM concentrations of 0, 10, or 25 µM media on Day 0. On Day 1, there was a full media change with POM, followed by half a media change on Day 4 with POM, and genomic DNA extraction of Day 7. **(B)** Neurons were transduced either in suspension like **(A)** or while adherent. For neurons transduced while adherent, on Day 0, cells were seeded, on Day 1, cells were transduced with virus and POM. On Day 4, media was changed and on Day 7, genomic DNA was extracted. Data represented in bar graphs are represented as mean +/- SEM, n = 5 technical replicates per condition, p-values generated by two-way ANOVA; ns = $p \geq 0.05$ (not shown), * for $p < 0.05$, ** for $p < 0.01$, *** for $p < 0.001$, **** for $p < 0.0001$).

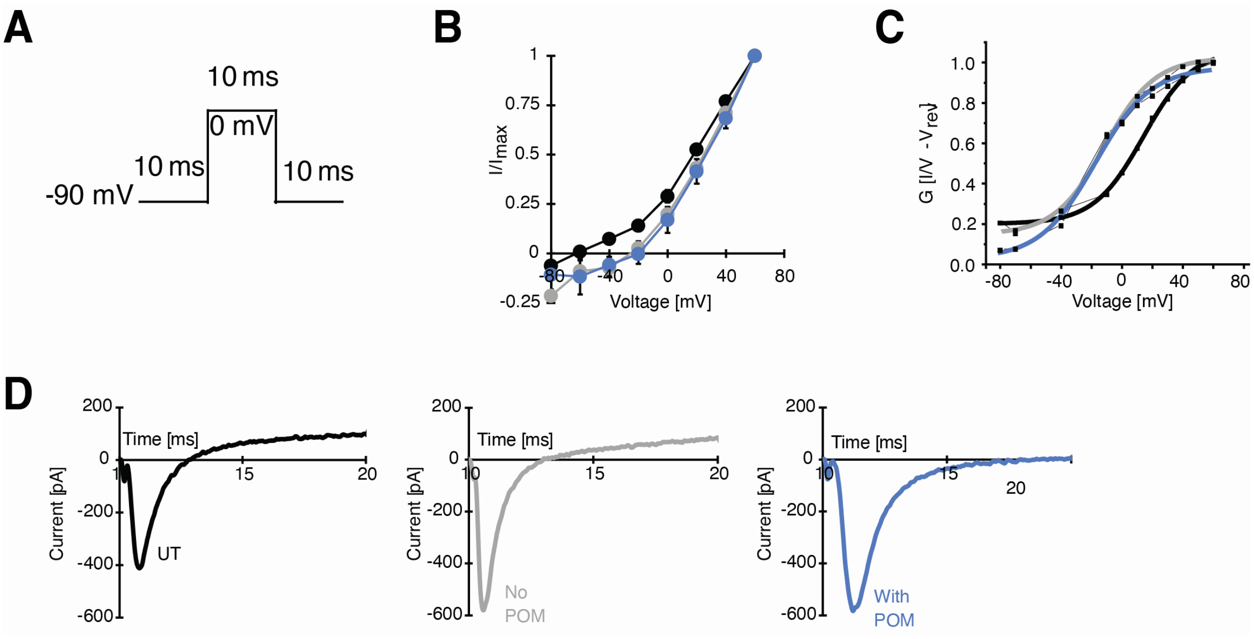

Figure S6. High-efficiency genome editing while preserving neuronal function in human hiPSC-derived cortical GABAergic neurons.

(A) Voltage response and Na current stimulation of a short (10 ms) hyperpolarizing current injection. **(B)** Normalized current (I/I_{max}), evoked by the same voltage steps (as shown in **Figure 5C**) in the neurons which were transduced with no POM (n=13), transduced with POM (n=12) and untreated (n=17). The error bar represents mean ± SE at each voltage. **(C)** Normalized conductance (G)-voltage plot in treated and untreated neurons. Solid line represents sigmoidal curve fit. **(D)** Na current in neurons, generated using a pulse protocol as shown in **A**. Untreated n = 17, No POM n = 13, POM n = 12.

Table S1. sgRNA targeting sequences.

	**sgRNA (5' - 3')**
AAVS1	GGGAACCCAGCGAGTGAAGA
VEGFA	GGGTGGGGGGAGTTTGCTCC
PCSK9	GGTGCTAGCCTTGCGTTCCG
APP	ATCCATTCATCATGGTGTGG

Table S2. PCR primers used for on- and off-target amplification of genomic loci for deep-sequencing.

	**Forward Primer Sequence (5' - 3')**	**Reverse Primer Sequence (5' - 3')**
AAVS1 On-Target	CTTCCTTCTCGGCGCTGCACCAC	AGCCAGGGAGACGGGGTACTTTGG
VEGFA On-Target	GCCCTGGGCTCTCTGTACAT	CACACCCCGGCTCTGGCTAA
PCSK9 On-Target	TGCTGCTGCTGCTCCTGGGT	GGAAGGTGGCTGTGGTTCCGTG
APP On-Target	GGTGTTGTCATAGCGACAGT	GCTAAGCCTAATTCTCTCA
VEGFA OTE1	ACTGCACCTGGCCATCATCC	TCTGTCCTATTTACACAGCAGACC
VEGFA OTE2	GTTCTGAGGCAGAGATGGCA	CATACAGGCCTGCTTCCTCC
VEGFA OTE3	TCCCCAGGGGACATTTGGCA	TGGGAGTTGTCCTGCAGCTGT

Table S3. Comparison of RNA expression of neo-substrates in different organs and cell types using Protein Atlas. The liver and kidney express high levels of the CRBN protein indicating it is sufficient for protein degradation to occur. Cell lines we used (HEK293T, Huh7, HepG2, inhibitory neurons) showed high levels of RNA expression for the CK1α and GSPT1 genes when compared to other genes. Data was not available for ARID2, SALL4, RNF166, and ZNF692 and protein expression data was not available for the eye (retina). (nTPM is transcripts per million)

Protein	What does it do?	Organ	Protein Expression	nTPM	Cell Type	nTPM	Reference
CRBN Cereblon	Plays a role in cellular and tissue metabolism, influences regulation of ion transport and AMP-activates protein kinase (AMPK) signaling pathway.	Cerebral cortex (brain) - neuronal cells	Medium	31.9	Inhibitory Neurons	70.9	Shi Q, Chen L, 2017
		Kidney	High	27.4	HEK293T	26.7	
		Liver - Hepatocytes	High	33.1	HepG2	16.6	
					Huh7	14.9	
		Eye (Retina)	No Data	21	hTERT-RPE1	32.8	
GSPT1 G1 to S phase transition 1	Translation termination factor crucial for releasing newly synthesized polypeptides from the ribosome by interacting with eukaryotic translation termination factor 1 (ETF1)	Cerebral cortex (brain) - neuronal cells	Medium	34.9	Inhibitory Neurons	45.1	NCBI Gene 2935
		Kidney	High	51.8	HEK293T	144.2	
		Liver - Hepatocytes	Medium	74	HepG2	281.3	
					Huh7	111.1	
		Eye (Retina)	No Data	34.2	hTERT-RPE1	118.1	
CSNK1A1 Casein kinase 1 alpha 1	Regulates cellular processes and signaling pathways	Cerebral cortex (brain) - neuronal cells	Not Detected	53.7	Inhibitory Neurons	110.5	Jiang S, et al., 2018
		Kidney	Low	48.5	HEK293T	73.2	
		Liver - Hepatocytes	Not detected	66.1	HepG2	110	
					Huh7	102.6	
		Eye (Retina)	No Data	55.9	hTERT-RPE1	158.1	
ZFP91 Zinc finger protein, atypical E3 ubiquitin ligase	Regulates the non-canonical NF-κB signaling pathway.	Cerebral cortex (brain) - neuronal cells	High	23.6	Inhibitory Neurons	0	Sun SC, 2010
		Kidney	High	24.2	HEK293T	22.8	
		Liver - Hepatocytes	High	29.7	HepG2	27	
					Huh7	23.8	
		Eye (Retina)	No Data	28.9	hTERT-RPE1	47.6	
	Terminates protein synthesis when interacting	Cerebral cortex (brain) - neuronal cells	Medium	10	Inhibitory Neurons	4.2	Betram G, et al. 2000

GSPT2 G1 to S phase transition 2	with eukaryotic release factor 1 (eRF1)	Kidney	Medium	6.1	HEK293T	11.9	
		Liver - Hepatocytes	Low	10.9	HepG2	0	
					Huh7	0	
		Eye (Retina)	No Data	7.5	hTERT-RPE1	13.1	
ARID2 AT-rich interaction domain 2	Regulates gene expression, chromatin organization, and cell differentiation.	Cerebral cortex (brain) - neuronal cells	No Data	3.8	Inhibitory Neurons	57.8	Bluemn T, et al., 2021
		Kidney	No Data	6.1	HEK293T	9.2	
		Liver - Hepatocytes	No Data	5.2	HepG2	10.2	
					Huh7	12	
		Eye (Retina)	No Data	12.8	hTERT-RPE1	12.9	
SALL4 Spalt like transcription factor 4	Transcription factor for maintaining stem cell pluripotency and self-renewal	Cerebral cortex (brain) - neuronal cells	No Data	0.1	Inhibitory Neurons	3.4	Tatestsu H, et al. 2016
		Kidney	No Data	0.8	HEK293T	0	
		Liver - Hepatocytes	No Data	1.5	HepG2	0.6	
					Huh7	1.8	
		Eye (Retina)	No Data	0.5	hTERT-RPE1	0	
RNF166 Ring finger protein 166	Mediates both Lys63-linked ubiquitination and sumoylation of its target proteins	Cerebral cortex (brain) - neuronal cells	No Data	18.7	Inhibitory Neurons	4.6	Hwang IY, et al., 2021
		Kidney	No Data	20.1	HEK293T	20.4	
		Liver - Hepatocytes	No Data	22.8	HepG2	23.4	
					Huh7	15.9	
		Eye (Retina)	No Data	3.3	hTERT-RPE1	6.6	
ZNF692 Zinc finger protein 692	Influences the shape of the nucleolus by interacting with nucleophosmin 1 (NPM1) and changing how it assembles itself	Cerebral cortex (brain) - neuronal cells	No Data	30.5	Inhibitory Neurons	18.9	Brown IN, et al., 2024
		Kidney	No Data	26.3	HEK293T	5.3	
		Liver - Hepatocytes	No Data	21.2	HepG2	4.4	
					Huh7	6.2	
		Eye (Retina)	No Data	8.2	hTERT-RPE1	5.6	
